# Comparison of FGM prevalence among Nigerian women aged 15–49 years using two household surveys conducted before and after the COVID-19 pandemic

**DOI:** 10.1186/s12889-024-19069-6

**Published:** 2024-07-12

**Authors:** Camille Morlighem, Corentin Visée, Chibuzor Christopher Nnanatu

**Affiliations:** 1 Department of Geography, University of Namur, 5000 Namur, Belgium; 2 ILEE, University of Namur, 5000 Namur, Belgium; 3 WorldPop, School of Geography and Environmental Science, University of Southampton, Southampton, SO17 1BJ UK; 4 Department of Statistics, Nnamdi Azikiwe University, PMB 5025, Awka, Nigeria

**Keywords:** FGM, Nigeria, Social norms, COVID-19, Bayesian spatial modelling

## Abstract

**Background:**

Due to its economic burden and change of focus, there is no gainsaying of the potential impacts of the COVID-19 pandemic on the progress of several female genital mutilation (FGM) interventions across the various countries. However, the magnitude of the potential changes in likelihood and prevalence should be more accurately explored and quantified using a statistically robust comparative study. In this study, we examined the differences in the likelihood and prevalence of FGM among 15-49 years old women before and after the pandemic in Nigeria.

**Methods:**

We used advanced Bayesian hierarchical models to analyse post-COVID-19 datasets provided by the Multiple Indicator Cluster Surveys (MICS 2021) and pre-COVID-19 data from the Demographic and Health Surveys (DHS 2018).

**Results:**

Results indicated that although there was an overall decline in FGM prevalence nationally, heterogeneities exist at state level and at individual-/community-level characteristics. There was a 6.9% increase in prevalence among women who would like FGM to continue within the community. FGM prevalence increased by 18.9% in Nasarawa, while in Kaduna there was nearly 40% decrease.

**Conclusions:**

Results show that FGM is still a social norm issue in Nigeria and that it may have been exacerbated by the COVID-19 pandemic. The methods, data and outputs from this study would serve to provide accurate statistical evidence required by policymakers for complete eradication of FGM.

**Supplementary Information:**

The online version contains supplementary material available at 10.1186/s12889-024-19069-6.

## Background

Female genital mutilation (FGM) is the partial or total removal of the external female genitalia for non-medical reasons. In addition to short-term harm, such as severe pain and shock, the practice has long-term consequences, including an increased risk of infertility, newborn deaths and urinary retention [1]. Often an ancestral practice passed down through generations, FGM is mostly performed on girls under the age of 15, based on ethnic and religious beliefs, as it is seen as a way to ensure purity before marriage [2]. The number of girls who have undergone FGM is estimated to be at least 200 million worldwide, with the majority in Africa, the Middle East, Asia and among immigrant communities in Western countries [3]. Considered a human rights violation and FGM elimination being one of the targets of the SDGs for 2030 (SDG 5, target 5.3), efforts in recent decades led by international and national organisations have succeeded in reducing the global prevalence of FGM among women and girls. Adolescent girls were a third less likely to be subjected to FGM in 2016 than 30 years earlier [4, 5]. However, with rapid population growth, this decline in prevalence has been accompanied by an increase in the absolute number of girls cut, and three million girls are still at risk of undergoing the practice each year [1].

Despite important efforts over the past decades, the COVID-19 pandemic is likely to have slowed or reversed progress against FGM practice, with the economic impact of the pandemic and the lockdown exacerbating violence against women and girls, including FGM, intimate partner violence and child abuse [6]. Evidence from previous research shows that the economic losses caused by the pandemic have led households to marry off their young daughters in exchange for a bride price, increasing FGM on girls [7, 8]. It has also led to the return of former cutters who had abandoned the practice, as well as new cutters entering the market, both as a strategy to earn an income [8]. School closures and home quarantines have also made girls more at risk of FGM by increasing the exposure of FGM victims to their perpetrators, while giving victims more time to recover before returning to school and avoiding the household to justify the girl's absence from school [8]. In addition, stress and economic insecurity, as well as difficulties in parenting, may have led to increased tension and violence in households, including towards children [7]. Furthermore, the COVID-19 outbreak led to a shift in focus for health systems and funding towards emergency response, affecting not only FGM but also broader public health issues such as tropical diseases [7–9]. As a result, FGM intervention activities and supports for FGM victims were disrupted and sometimes stopped during the pandemic [10].

Among FGM-practicing countries, Nigeria is one of the countries with the highest prevalence of FGM [11]. Due to its large population, Nigeria has the highest absolute number of cut women and girls in the world, with an estimated 19.9 million women and girls cut between 2004 and 2015 [12]. In response to the SDG target, Nigeria passed a federal law, the Violence against Persons (Prohibition) Act 2015 (VAPP Act), which prohibits any form of gender-based violence, including FGM, with consequences for the perpetrator [13]. Previous research has examined spatio-temporal trends in FGM prevalence in Nigeria by combining multiple datasets from the Demographic and Health Surveys (DHS) and Multiple Indicator Cluster Surveys (MICS) [11, 14]. The prevalence of FGM among Nigerian women aged 15–49 decreased from 29.6% in 2008 to 18.4% in 2017, while the prevalence among girls aged 0–14 decreased from 30.0% to 25.3% over the same period [11]. However, while the prevalence of FGM among women aged 15 – 49 years has decreased at the national level, there were geographical variations in prevalence at the state levels. For example, while decreasing in Nigeria's southeastern states, prevalence of FGM increased in the northwestern states of the country from almost zero in 2003 to 39.3% in 2017 [11]. In recent years, the COVID-19 pandemic may have affected the progress made in reducing the prevalence of FGM among women and girls in Nigeria, with potentially different effects in the southern and northern states of the country.

Previous research has highlighted the perceived impact of the pandemic on the practice of FGM through surveys within the population and programme implementers in FGM-practicing countries [7–10, 15]. However, there is a notable lack of work examining trends in FGM prevalence and potential spatio-temporal patterns over this period using statistical evidence data. Other studies have shown that advanced statistical techniques, such as Bayesian hierarchical models, can provide significant insights into the role of key determinants of FGM, while accounting for spatial random variation [11, 14, 16–19]. Therefore, the aim of this study is to compare the prevalence and likelihood of FGM among Nigerian women aged 15–49 years before and after the COVID-19 pandemic, with respect to individual (e.g., a woman's marital status, wealth quintile, education, age) and community level (e.g., the proportion of circumcised women in the community, the proportion of women who support the continuation of FGM in the community) determinants of FGM practice and a woman's state/zone of residence. We used data from the DHS conducted in Nigeria in 2018, referred to as the pre-COVID-19 pandemic period, and the MICS conducted in Nigeria in 2021, referred to as the post-pandemic period. Using Bayesian hierarchical models, we examined spatial and temporal patterns of FGM practice across Nigeria's 36 states and the Federal Capital Territory (FCT).

## Methods

### Data

**Fig. 1:**
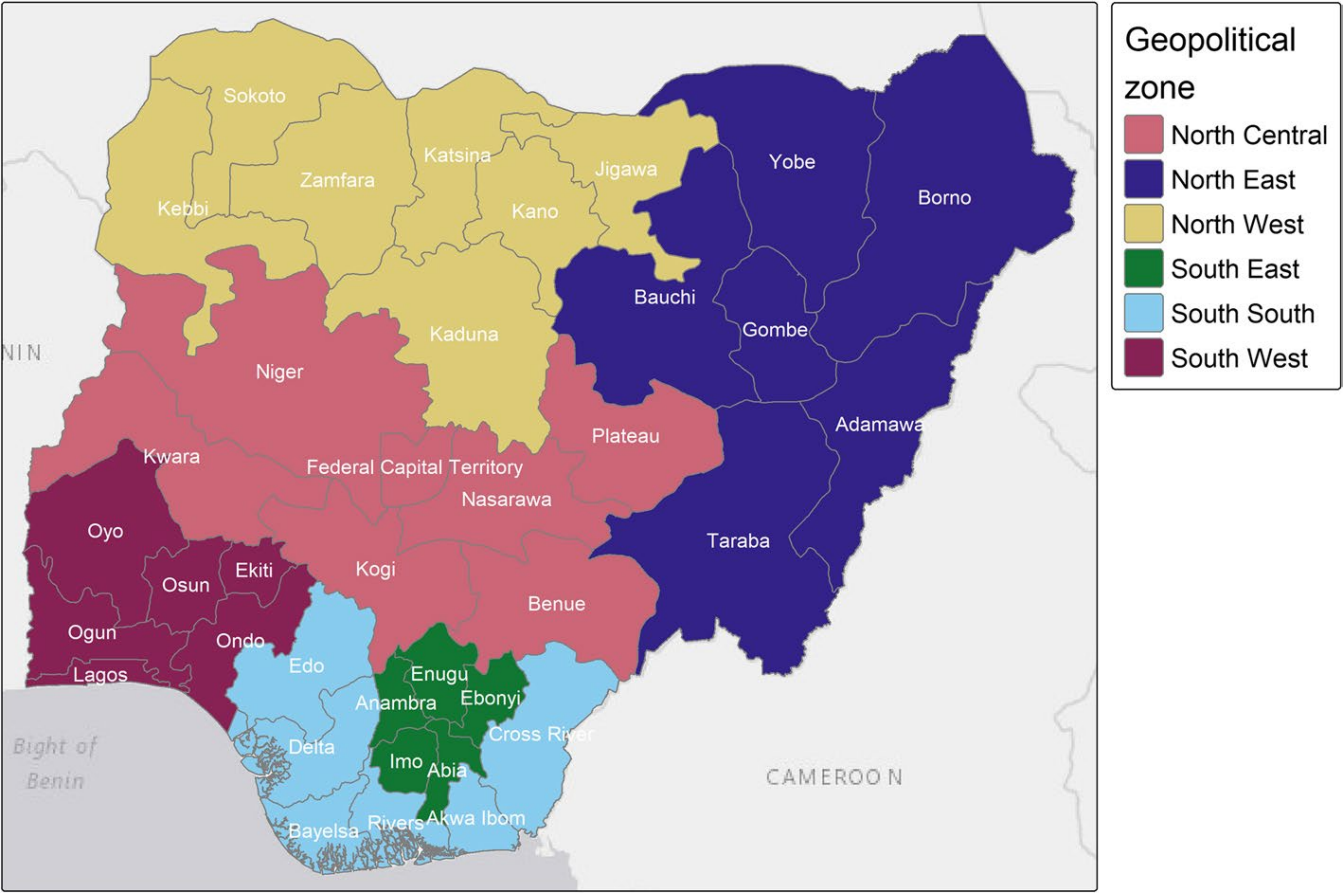
The 36 Nigerian states and the Federal Capital Territory in the six geopolitical zones. Shapefile was downloaded from GADM

FGM prevalence data for Nigeria were extracted from the 2018 DHS (pre-COVID-19 period) and the 2021 MICS (post-COVID-19 period). Both surveys are very similar in terms of sampling strategy and sample composition. The sampling frame is based on a two-stage stratified sampling design which was implemented by first selecting clusters as primary sampling units across all 36 Nigerian states and the FCT (Fig. 1), and then randomly selecting households within the clusters. All eligible women within the selected households that are aged 15–49 were asked in the Women's Questionnaire whether they have ever heard of FGM and, if so, whether they have ever undergone FGM and their opinion on the continuation of the practice. In the 2018 DHS, 41821 women were interviewed in 1400 clusters [20], while in the 2021 MICS, 40326 women were interviewed in 1755 clusters [21].

### Outcome and exposure variables

The outcome variable in this study is the FGM status of a woman, a binary variable coded 1 if the woman has been cut at the time of the survey and 0 if not. We relate a woman's FGM status to individual and community level (i.e. cluster level) exposure variables, as well as the region and state of residence of the woman. Variables indicative of FGM as a socio-cultural norm included the percentage of women cut in the community, the percentage of women in the community who support the continuation of FGM, and the woman's support for the continuation of FGM. In addition, socio-demographic variables at the individual level included the woman's type of residence (urban vs rural), age, ethnicity, religion, marital status, wealth quintile and the woman's level of education. Due to differences in data collection, the DHS religion and ethnicity variables are based on women's individual responses, whereas the MICS religion and ethnicity are based on the household head. Additional socio-demographic variables aggregated at the community level included the main religion in the community, the most represented wealth quintile, and an ethnic fractionalisation index (EFI) introduced in [18].

The EFI is a continuous variable that measures the degree of ethnic heterogeneity within a community. It is calculated as follows:

1 $$EFI=1-\sum_{k=1}^{n}{s}_{k}^{2}$$

Where ${s}_{k}$ is the proportion of the ${k}^{th}$ ethnic group in a community with $n\ge 2$ ethnic groups. This variable ranges from 0 to 1, with values close to 1 indicating a multi-ethnic community where ethnic groups are of comparable size, and values close to 0 indicating a community with fewer ethnic groups. The EFI assumes that in a multi-ethnic community, it may be easier to move towards ending the practice of FGM if one or more ethnic groups support this change, whereas in a mono-ethnic community that supports the practice of FGM, it may be more difficult to make such a change [18].

### Bayesian regression models

In this paper, building on previous work, we used a Bayesian logistic regression to model the likelihood for a woman to be cut as a function of the set of individual and community level variables defined above. The Bayesian framework allows us to provide uncertainty in the final estimates of FGM prevalence and leveraging spatial information. Bayesian models were implemented under the integrated nested Laplace approximation (INLA) framework [22], within the R-INLA package, which offers significant improvements in computational requirement compared to the classical Markov chain Monte Carlo (MCMC) approaches [22].

Consider ${y}_{i}$ as the FGM status of woman $i$, such that ${y}_{i}$ is one (1) when the woman was cut or zero (0) when she was not. The random variable ${y}_{i}$ follows a Bernoulli distribution with probability ${p}_{i}$ for a woman $i$ to be cut. The model is further expressed as follows:

2 $$logit\left({p}_{i}\right)= {\beta }_{0}+{{z}_{i}^{\prime}\beta }+{f}_{1}\left({x}_{i1}\right)+\dots +{{f}_{p}\left({x}_{ip}\right)+f}_{spat}\left({s}_{i}\right)$$

Where ${\beta }_{0}$ is the intercept, ${z}_{i}^{\prime}$ is the vector of covariates with regression coefficients $\beta$ and ${f}_{1}\left(.\right),\dots ,{f}_{p}\left(.\right)$ are the smooth functions of non-linear covariates, ${x}_{i1,\dots ,p}$ such as age or the prevalence of FGM in the community, as done in [14, 18]. ${f}_{spat}\left({s}_{i}\right)$ is the spatial random variation at ${s}_{i}\in \left\{1,\dots , 37\right\}$, the state of residence of woman $i$ among the 36 Nigerian states and the FCT. ${f}_{spat}\left({s}_{i}\right)$ can be further decomposed as:

3 $${f}_{spat}\left({s}_{i}\right)={f}_{str}\left({s}_{i}\right)+{f}_{unstr}\left({s}_{i}\right)$$

${f}_{str}\left({s}_{i}\right)$ is the structured or correlated spatial variation, that allows to account for the spatial autocorrelation between neighbouring states, assuming, based on Tobler’s first law of Geography [23], that two states that are close to each other (i.e. neighbours) are more likely to have similar response values. From this it can be assumed that states that are further apart are spatially independent of each other and are not correlated; this is the remaining spatial variation. This spatial heterogeneity between non-neighbouring states is accounted for by ${f}_{unstr}\left({s}_{i}\right)$, which represents the unstructured or uncorrelated spatial variation.

The intercept ${\beta }_{0}$ is assigned a Gaussian prior with mean and precision equal to zero (${\beta }_{0}\sim N(\text{0,0}$)) and the regression coefficients $\beta$ are assigned a Gaussian prior with zero mean and precision 0.001, which are the default priors of R-INLA. Non-linear covariate effects modelled using smooth functions ${f}_{1}\left(.\right),\dots ,{f}_{p}\left(.\right)$ are assigned an independent and identically distributed (i.i.d.) Gaussian prior such that $\left.{f}_{l}\left(.\right)\right|{\tau }_{l}\sim N\left(0,\frac{1}{{\tau }_{l}}\right)$, where $l\in \left\{1,\dots , p\right\}\text{ and }{\tau }_{l}$ is a precision parameter. The structured spatial effects ${f}_{str}\left({s}_{i}\right)$ are modelled using an intrinsic conditional autoregressive (iCAR) model of type *Besag* [24], where the values ${u}_{j}$ of a collection of states $j \in \left\{1,\dots , 37\right\}$ depends on the neighbouring states as follows [25]:

4 $$\left.{u}_{j}\right|{u}_{-j}, {\tau }_{s}\sim N\left(\frac{1}{{d}_{j}}\sum_{k\sim j}{u}_{k},\frac{1}{{d}_{j}}\frac{1}{{\tau }_{s}}\right)$$

Where $k\sim j$ denotes that state $k$ and $j$ are neighbours, ${d}_{j}$ is the number of neighbours and ${\tau }_{s}$ is the precision parameter that controls the amount of variation between the neighbouring states. Neighbourhood between states is defined based on a binary adjacency matrix, where two states are considered neighbours if they share at least one point along their common boundary [26]. The unstructured spatial effects ${f}_{unstr}\left({s}_{i}\right)$ are modelled using a zero-mean i.i.d. Gaussian prior such that:

5 $$\left.{f}_{unstr}\left(s\right)\right|{\tau }_{u}\sim N\left(0,\frac{1}{{\tau }_{u}}\right)$$

Where ${\tau }_{u}$ is a precision parameter. Precision parameters ${\tau }_{j}$, $j$ being a generic term for $l, u, s$, are assigned log-gamma hyperpriors with rate and scale parameters of 1 and 5e-05.

Based on different combinations of spatial random effects in (2), four different model structures were tested: (1) a Base model with an intercept-term and covariates, (2) an IID model, which is the Base model with uncorrelated spatial random effects, (3) a Besag model, which is the Base model with correlated spatial random effects, and (4) an IID + Besag model, which is the Base model with both correlated and uncorrelated spatial random effects. These model structures are summarised in Table 1. In addition, to assess how individual and community level factors influence the likelihood of FGM, we fitted three different sets of covariates for each model structure: (1) the first set included all individual level variables, (2) the second set included all community level variables, and (3) the third set included a mixture of individual and community level variables (Table 2). To adjust for sample representativeness, all models included the survey sampling weights as a covariate. We used Deviance Information Criterion (DIC) to identify the model structure that best fits the data (i.e. the model that minimises DIC). We further compared models based on (1) individual, (2) community, and (3) individual and community level variables using the ${R}^{2}$, Root Mean Square Error (RMSE), and Mean Absolute Error (MAE) calculated on the observed and posterior predicted FGM prevalence per state. Model estimates are presented as posterior odd ratios (POR).

**Table 1:** Model structures

Model	Structure	Description	Complexity
Base	${\beta }_{0}+{{z}_{i}^{\prime}\beta }+{f}_{1}\left({x}_{i1}\right)+\dots +{f}_{p}\left({x}_{ip}\right)$	Intercept + covariates	1
IID	${\beta }_{0}+{{z}_{i}^{\prime}\beta }+{f}_{1}\left({x}_{i1}\right)+\dots +{f}_{p}\left({x}_{ip}\right)+{f}_{unstr}\left({s}_{i}\right)$	Base + uncorrelated spatial RE	2
Besag	${\beta }_{0}+{{z}_{i}^{\prime}\beta +}{f}_{1}\left({x}_{i1}\right)+\dots +{f}_{p}\left({x}_{ip}\right)+{f}_{str}\left({s}_{i}\right)$	Base + correlated spatial RE	3
IID + Besag	${\beta }_{0}+{{z}_{i}^{\prime}\beta }+{{f}_{1}\left({x}_{i1}\right)+\dots +{f}_{p}\left({x}_{ip}\right)+f}_{str}\left({s}_{i}\right)+{f}_{unstr}\left({s}_{i}\right)$	Base + correlated spatial RE + uncorrelated spatial RE	4

The complexity column ranks the complexity of the model from 1 to 4, where 1 is the simplest model and 4 is the most complex. RE stands for random effects

**Table 2:** Combination of individual and community level variables fitted in the models

Level	Variables
Individual	Geopolitical zone, residence, education, age, wealth quintile, marital status, ethnicity, religion, support for FGM continuation
Community	Percentage of women supporting FGM continuation, percentage of women that are cut, EFI, main religion in community, main wealth quintile in community
Individual & community	Geopolitical zone, residence, education, age, wealth quintile, marital status, percentage of women supporting FGM continuation, percentage of women that are cut, EFI, main religion in community

## Results

### Descriptive analysis

**Fig. 2:**
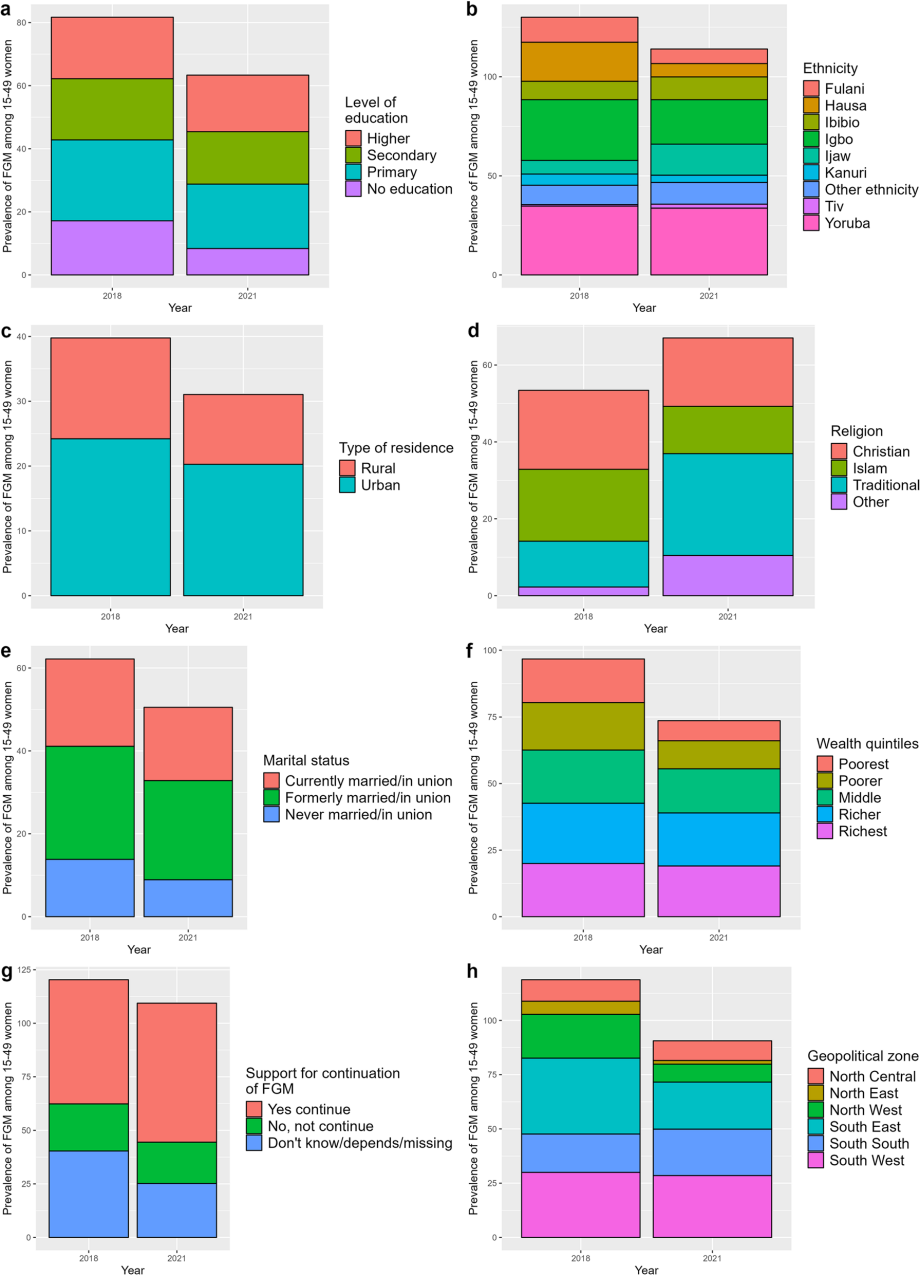
FGM prevalence by some individual and community level characteristics in 2018 (DHS) and 2021 (MICS)

The national prevalence of FGM, calculated from survey data, decreased from 19.5% in 2018 (DHS) to 15.1% in 2021 (MICS). However, the patterns of change in FGM prevalence are scale and group-dependent, as FGM prevalence varied by geographic location, level of education, ethnicity, and religion as well as other socio-economic and socio-demographic characteristics (Fig. 2 and Table 3).

**Table 3:** FGM prevalence by some individual and community level characteristics in 2018 (DHS) and 2021 (MICS)

Variable	Levels	DHS 2018 (%)	MICS 2021 (%)
Geopolitical zone	North-Central	9.9	9.1
	North-East	6.1	1.7
	North-West	20.2	8.2
	South-East	35.0	21.7
	South-South	17.7	21.4
	South-West	30.0	28.5
Education	Higher	19.5	17.9
	Secondary	19.4	16.6
	No education	17.2	8.4
	Primary	25.6	20.4
Ethnicity	Igbo	30.7	22.4
	Tiv	0.8	1.9
	Other ethnicity	9.8	11.0
	Hausa	19.7	6.7
	Ijaw	6.9	15.6
	Yoruba	34.7	33.7
	Fulani	12.6	7.3
	Kanuri	5.6	3.6
	Ibibio	9.3	11.5
Marital status	Currently married/in union	21.1	17.7
	Never married/in union	13.8	8.9
	Formerly married/in union	27.3	23.9
Residence	Urban	24.2	20.2
	Rural	15.6	10.8
Wealth quintile	Richest	20.0	19.0
	Richer	22.6	19.9
	Middle	20.0	16.5
	Poorer	17.8	10.5
	Poorest	16.4	7.5
Religion	Christian	20.6	17.8
	Islam	18.7	12.3
	Traditional	11.9	26.5
	Other	2.2	10.4
Support for FGM continuation	No, not continue	22.0	19.2
	Yes continue	58.0	64.9
	Don't know/depends/missing	40.4	25.2

**Fig. 3:**
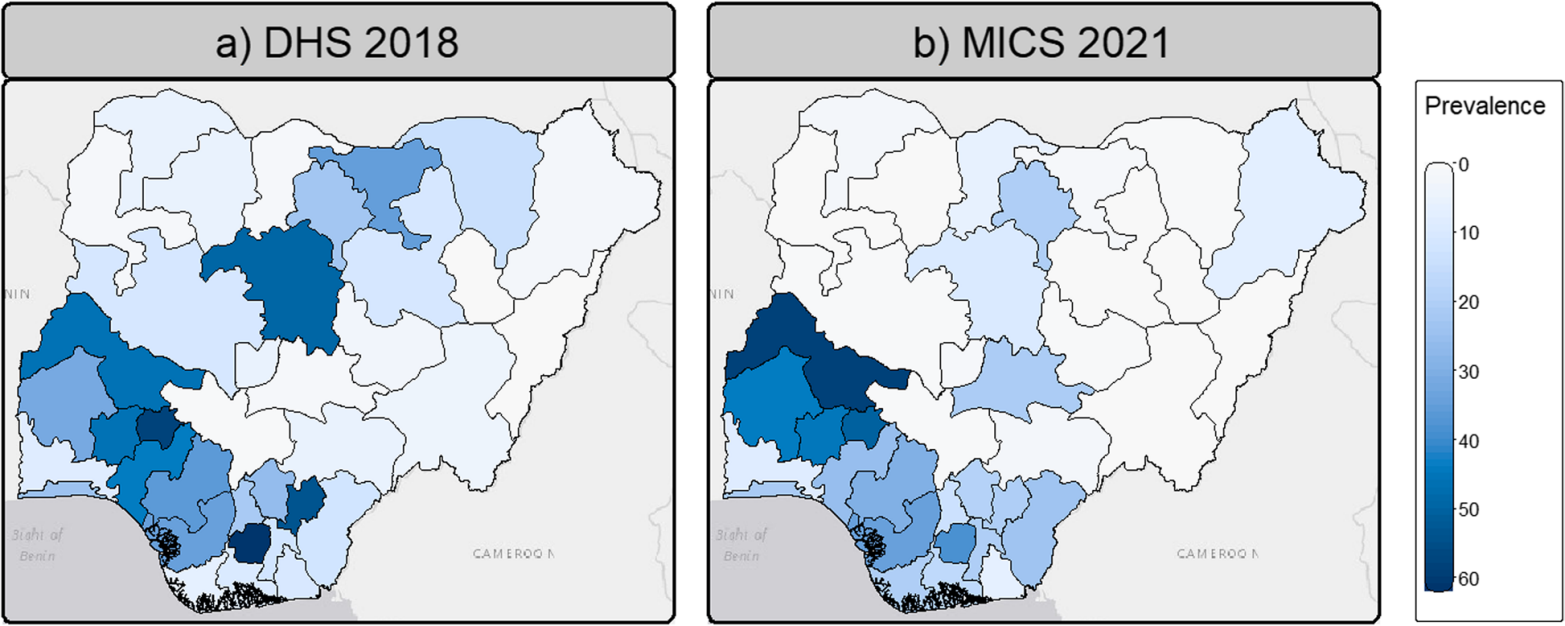
FGM prevalence in Nigerian states and FCT in 2018 (DHS) (**a**) and 2021 (MICS) (**b**). Shapefile downloaded from GADM

The decrease in FGM prevalence was also observed in most Nigeria's geopolitical zones (see Table 3). FGM prevalence decreased in all Northern zones, with a particularly significant decrease in the North-West zone, decreasing from 20.2% to 8.2% between 2018 and 2021. FGM prevalence also declined in most Southern zones, particularly in the South-East zone, from 35.0% to 21.7%, but increased in the South-South zone from 17.7% in 2018 to 21.4% in 2021 (see Table 3). These spatial trends in FGM prevalence further mask some heterogeneity when zooming down to the state level (Fig. 3 and Table S1). FGM prevalence decreased in most northern states between 2018 and 2021, with decreases of more than 30% in both Jigawa and Kaduna. In the North-Central zone, while FGM prevalence decreased in Niger and FCT, it increased in Kwara and Nasarawa states by 12% and almost 20% respectively between 2018 and 2021. In the south, Ebonyi and Imo, two of the states with the highest prevalence of FGM in 2018, saw a significant decrease in prevalence from 53.2% to 20.4% and 61.72% to 37.93% in 2021 respectively. However, neighbouring southern states such as Abia, Rivers and Cross River showed a different pattern, with FGM prevalence increasing between 2018 and 2021, up to an increase of more than 10% in Cross River. Also in the south, FGM prevalence increased in Bayelsa.

In terms of educational attainment, the decline in FGM prevalence was most pronounced in the “no education” group, falling from 17.2% to 8.4%, while there was little change in the “higher education” group (Table 3). While the practice of FGM generally decreased among different ethnic groups between 2018 and 2021, particularly among the Hausa (i.e. 19.7% in 2018 to 6.7% in 2021), it remained high among the Yoruba (i.e. 34.7% to 33.7%) and increased among the Tiv (i.e. 0.8% to 1.9%), Ijaw (i.e. 6.9% to 15.6%) and Ibibio (i.e. 9.3% to 11.5%). FGM prevalence also increased among traditionalists, from 11.9% in 2018 to 26.5% in 2021, making them the main group performing FGM in 2021, ahead of Muslims and Christians (Table 3).

The prevalence of FGM decreased across all marital statuses, with a greater decrease among never married/in union women than among currently married/in union and formerly married/in union. By household wealth, most of the progress in FGM prevalence has been made in the poorest and poorer wealth quintiles, with FGM prevalence decreasing from 16.4% to 7.5% and 17.8% to 10.5%, respectively, over the 2018–2021 period. Finally, the prevalence of FGM has decreased from 22.0% to 19.2% over the 2018–2021 period among women who support the abandonment of FGM, while it has increased from 58.0% to 64.9% among women who support the continuation of FGM.

### Bayesian regression models

#### Model fit indices

##### DIC

The Deviance Information Criterion (DIC) [27] was used for the model selection such that models with lower DIC values are retained as the best fit models. DICs of the three Bayesian regression models (i.e. with individual level variables, with community level variables, and with both individual and community level variables) tested with different model structures are shown in Table 4..

**Table 4.** Comparison of model structure for Bayesian regression models using DIC

	**DHS 2018**			**MICS 2021**		
**Model**	**Individual**	**Community**	**Individual & community**	**Individual**	**Community**	**Individual & community**
Base	14,023	12,081	11,755	17,673	14,444	13,193
IID	**12,347**	**11,995**	11,701	**15,678**	14,412	13,189
Besag	**12,347**	11,998	**11,700**	**15,678**	**14,407**	13,190
IID + Besag	**12,347**	**11,995**	11,702	**15,678**	14,408	**13,188**

For each set of variables, i.e. individual, community and individual & community level variables, the model with the lowest DIC is shown in bold, indicating best model fits. However, note that DIC differences of less than 2 are not significant [27]

Adding spatial random effects (whether correlated or uncorrelated) to the Base model improves model fit for all combinations of individual/community level variables, as all three IID, Besag and IID + Besag models always yield lower DIC values (see Table 4.). This means that accounting for spatial autocorrelation between neighbouring states (i.e. via the Besag model) and/or residual uncorrelated spatial variation between non-neighbouring states (i.e. via the IID model) improves model fit compared to the Base model with covariates only. However, when comparing the spatial models together, given that DIC differences of less than 2 are not significant [27], there are no significant differences between the IID, Besag and IID + Besag models for any combination of variables, except for the community level model fitted to MICS data, where the Besag model outperforms the IID model (Table 4.). Overall, the best-fitting models are spatial models that include both individual and community level variables for both DHS 2018 and MICS 2021. For the sake of parsimony, simpler models should be preferred when the DIC difference is less than 2 [27], hence we retained simpler model structures (see the complexity rank in Table 1) for the next validation exercise when the difference in DIC met this criterion.

##### ${R}^{2}$, RMSE and MAE

In addition, we carried out further model validation that tested the predictive performances of the various models. In particular, we used a constellation of model fit metrics including ${R}^{2}$, Root Mean Square Error (RMSE), and Mean Absolute Error (MAE). The results of the performance metrics on the various models are given in Table 5. These metrics are calculated based on the observed FGM prevalence and the predicted posterior FGM prevalence across the models at the state level. For both DHS and MICS data, the individual level model was outperformed by the other two models on all performance metrics. The model using both individual and community level variables then slightly improved the predictive performance compared to using only community level variables, with an ${R}^{2}$ of 0.95 for DHS and 0.92 for MICS. This model was then used for all subsequent analyses in this paper, with the IID model structure. Posterior estimates based on the other model structures (Base, Besag, IID + Besag) are provided in the supplementary information (see Tables S2-S4 and Figs. S1-S6). These additional results demonstrate the close similarity between the results of the IID, Besag and IID + Besag models, and thus support the decision to use parsimony.

**Table 5:** Comparison of model predictive performance using ${R}^{2}$, RMSE and MAE

	**DHS 2018**			**MICS 2021**		
**Metric**	**Individual (IID)**	**Community (IID)**	**Individual & community (IID)**	**Individual (IID)**	**Community (Besag)**	**Individual & community (IID)**
${R}^{2}$	0.88	**0.95**	**0.95**	0.78	**0.92**	**0.92**
RMSE	16.78	10.69	**10.26**	15.24	8.25	**7.48**
MAE	14.07	8.23	**7.83**	11.96	5.57	**4.97**

Models with the lowest RMSE, MAE and highest ${R}^{2}$ are shown in bold, indicating the best model performance. RMSE, MAE and ${R}^{2}$ values are calculated by comparing the observed and posterior predicted FGM prevalence per state

#### Posterior odd ratios

To assess changes in the likelihood of FGM, we calculated the posterior odds ratios (POR) of the best performing model (i.e. the model with the lowest RMSE and MAE values and the highest ${R}^{2}$). The POR is obtained by exponentiating the posterior fixed effects estimate of the model, and the results obtained from the IID models with individual and community level variables for both DHS and MICS are presented in Table 6. While some variables have a significant effect on women's FGM status in both 2018 and 2021, others are only significant for one period. Some variables also show different effects depending on the period considered. These are discussed in more detail in the following sections.

**Table 6:** Posterior odd ratios from the Bayesian models fitted to DHS 2018 and MICS 2021 data

Variables	Levels	DHS 2018			MICS 2021		
		**POR**	**2.5%**	**97.5%**	**POR**	**2.5%**	**97.5%**
	(Intercept)	**2.033**	**1.239**	**3.326**	1.483	0.972	2.244
**Geopolitical zone**	North–North (ref)	1	-	-	1	-	-
	North-East	**0.398**	**0.211**	**0.754**	**0.459**	**0.265**	**0.797**
	North-West	0.715	0.410	1.258	1.368	0.944	2.038
	South-East	0.783	0.432	1.438	0.739	0.521	1.071
	South-South	0.789	0.449	1.396	0.774	0.553	1.105
	South-West	1.220	0.701	2.152	0.993	0.716	1.418
**Residence**	Rural (ref)	1	-	-	1	-	-
	Urban	1.001	0.862	1.162	**1.173**	**1.009**	**1.363**
**Education**	No education (ref)	1	-	-	1	-	-
	Higher	**0.584**	**0.471**	**0.725**	**0.669**	**0.544**	**0.822**
	Primary	1.120	0.946	1.327	1.052	0.881	1.256
	Secondary	**0.781**	**0.658**	**0.927**	**0.791**	**0.665**	**0.941**
**Age**		*See *Fig. 4a			*See *Fig. 4b		
**Wealth quintile**	Poorest (ref)	1	-	-	1	-	-
	Poorer	0.908	0.758	1.086	0.893	0.748	1.065
	Middle	0.852	0.700	1.038	0.865	0.720	1.038
	Richer	0.817	0.659	1.013	**0.749**	**0.615**	**0.912**
	Richest	0.859	0.677	1.090	**0.594**	**0.477**	**0.740**
**Marital status**	Currently married/in union (ref)	1	-	-	1	-	-
	Formerly married/in union	**1.437**	**1.178**	**1.751**	1.055	0.899	1.239
	Never married/in union	**0.653**	**0.564**	**0.758**	**0.597**	**0.516**	**0.690**
**Percentage women cut**		*See *Fig. 4c			*See *Fig. 4d		
**Percentage women supporting FGM continuation**		*See *Fig. 4e			*See *Fig. 4f		
**EFI**		**0.618**	**0.438**	**0.873**	0.817	0.597	1.119
**Main religion in community**	Christian (ref)	1	-	-	1	-	-
	Islam	0.952	0.764	1.186	1.096	0.902	1.330
	Traditional	0.565	0.058	5.509	0.000	0.000	1.899
**Sampling weight**		0.986	0.896	1.085	0.996	0.951	1.042

Posterior odd ratios (POR) estimates are based on the IID models using both individual and community level variables for both DHS 2018 and MICS 2021. Figures in bold indicate significant relationships, i.e. when the 2.5% and 97.5% CIs are both either greater or less than 1

**Fig. 4:**
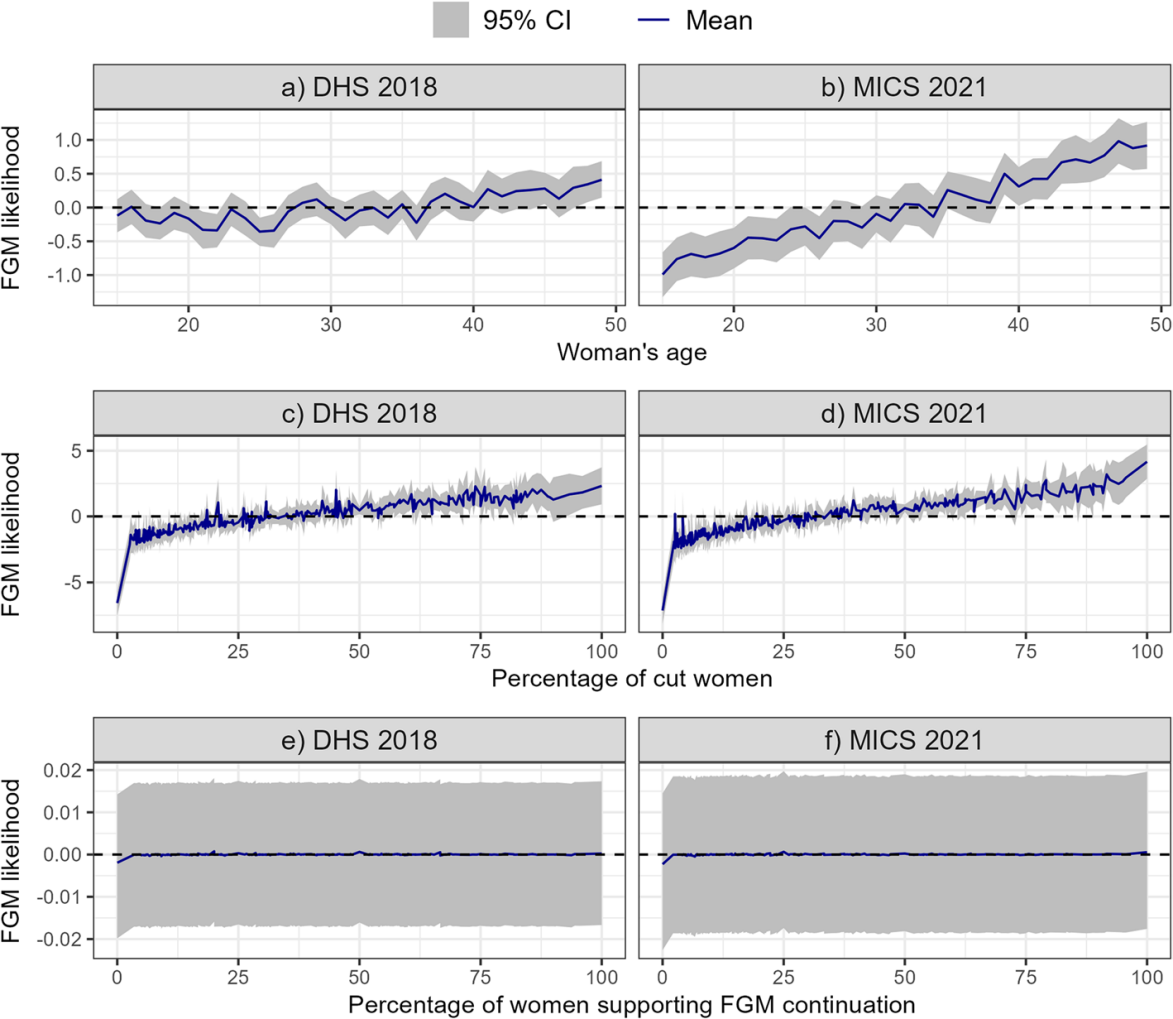
Non-linear effects of woman’s age (**a**, **b**), percentage cut (**c**, **d**) and women supporting FGM continuation (**e**, **f**). Estimates are based on the IID models using both individual and community level variables for both DHS 2018 and MICS 2021

In terms of the location of individuals in the country's geopolitical zones, women in the North-East are more than twice as likely to be cut than women living in the North–North (the reference group) in both 2018 and 2021. However, there is no significant difference in the likelihood of FGM across all other geopolitical zones. In 2021, living in an urban area is significantly associated with an increased likelihood of FGM compared to living in a rural area. Educational attainment is another key factor at the individual level in determining the likelihood of a woman undergoing FGM; in both 2018 and 2021, the likelihood of FGM is lower for women with secondary and higher education compared to women with no education. While some variables, such as educational attainment, show a constant effect on the likelihood of FGM in both 2018 and 2021, others show interesting changes over time, such as marital status. While being formerly married increases the likelihood of FGM by almost 50% in 2018 compared to women who are currently committed, being never married is always associated with a lower likelihood of FGM, even more so in 2021 than in 2018. Household wealth does not strongly affect the likelihood of FGM; only women from the richer and richest wealth quintiles are significantly less likely to be cut in 2021 than women from the poorest wealth quintile. Finally, at the individual level, Fig. 4a and b show that the likelihood of a woman having undergone FGM increases with her age, and the slope of the increase is even steeper in 2021 than in 2018.

At the community level, the likelihood of FGM increases with the proportion of cut women in the community in both 2018 and 2021, with a steeper increase in FGM likelihood in 2021 when FGM prevalence in the community exceeds 75% (Fig. 4c and d). However, Fig. 4e and f show that there is no clear effect of the percentage of women who support the continuation of FGM on the likelihood of FGM. Another key FGM indicator related to socio-cultural norms is the EFI, with a significantly lower likelihood of FGM found in multi-ethnic communities (i.e. with higher EFI scores) in 2018.

#### Posterior estimates of FGM prevalence

**Fig. 5:**
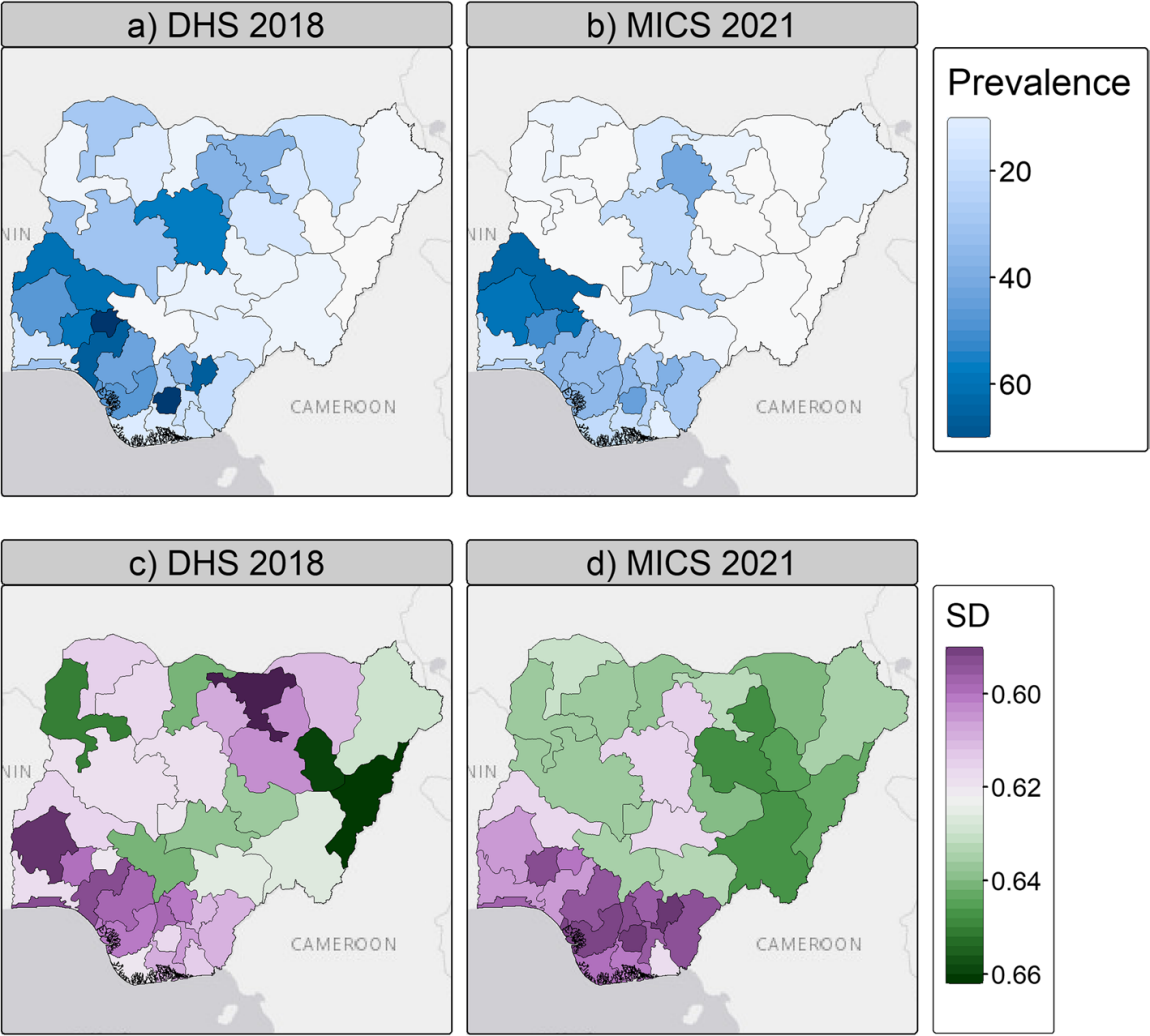
Posterior predicted FGM prevalence among women aged 15–49 years (**a**, **b**) and uncertainty (**c**, **d**) estimates. Posterior estimates are based on the IID models using both individual and community level variables for both DHS 2018 and MICS 2021. SD stands for standard deviation. Shapefile downloaded from GADM

**Fig. 6:**
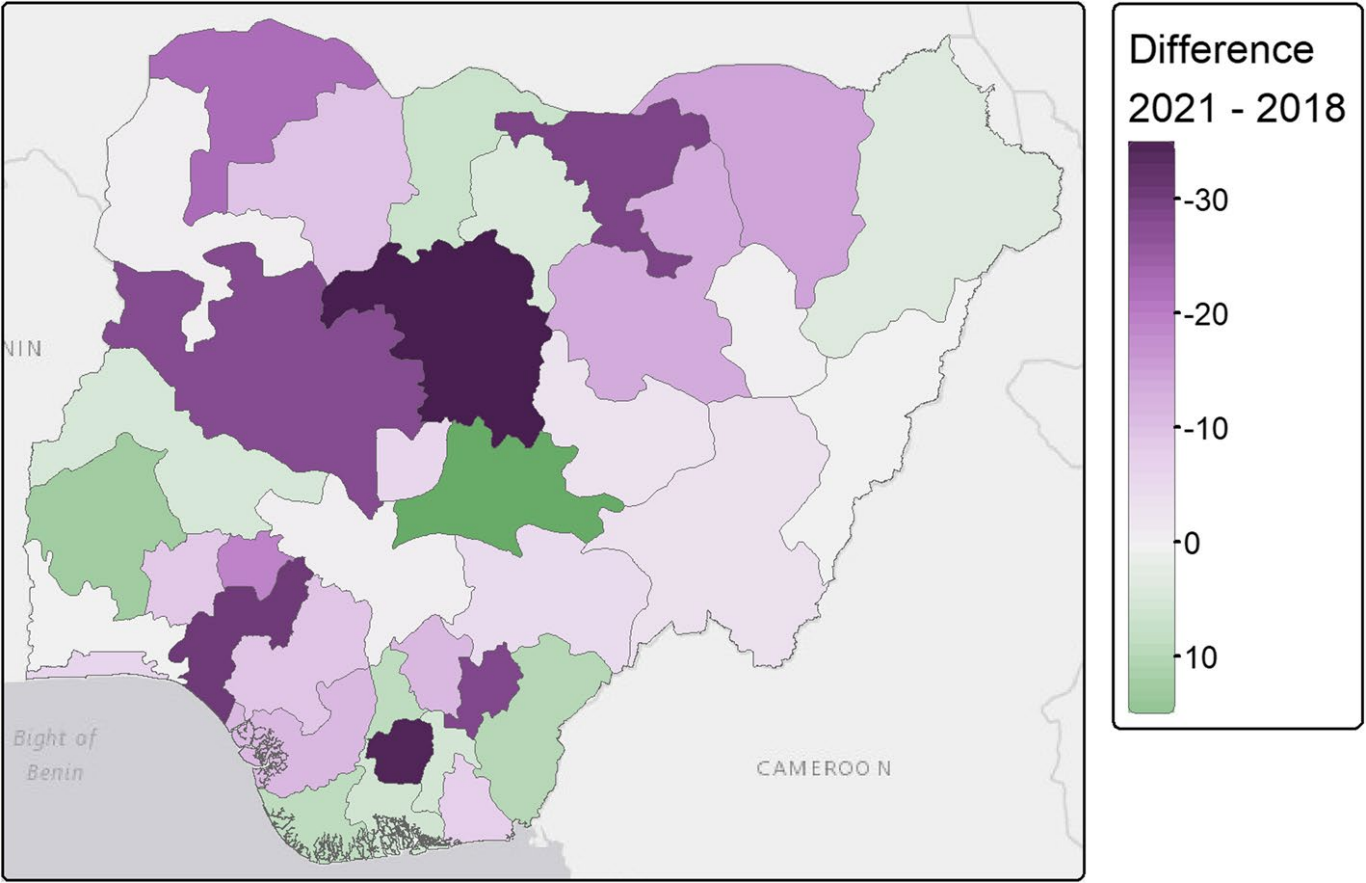
Difference in the posterior predicted FGM prevalence per state between 2021 (MICS) and 2018 (DHS). Green areas indicate that the FGM prevalence was higher in 2021 than in 2018, while purple areas indicate that the FGM prevalence has decreased over the period. Posterior estimates are based on the IID models using both individual and community level variables for both DHS 2018 and MICS 2021

The predicted national prevalence of FGM is 25.6% in 2018 (DHS), falling to 17.3% in 2021 (MICS). This is 6.1% and 2.2% higher than the observed prevalence in the DHS and MICS respectively. The maps of predicted FGM prevalence at the state level (Fig. 5a and b) are consistent with the maps of observed prevalence (Fig. 3), with an overall decrease in northern Nigerian states between 2018 and 2021, but an increase in some southern states such as Oyo and Abia and in Nasarawa and Kwara states in the North-Central zone. Figure 6 further highlights that high heterogeneities exist between states and their evolution between 2018 and 2021 regarding FGM prevalence. In 2018, the highest predicted prevalence of FGM is in Ekiti state, while in 2021 it is in Kwara state.

**Fig. 7:**
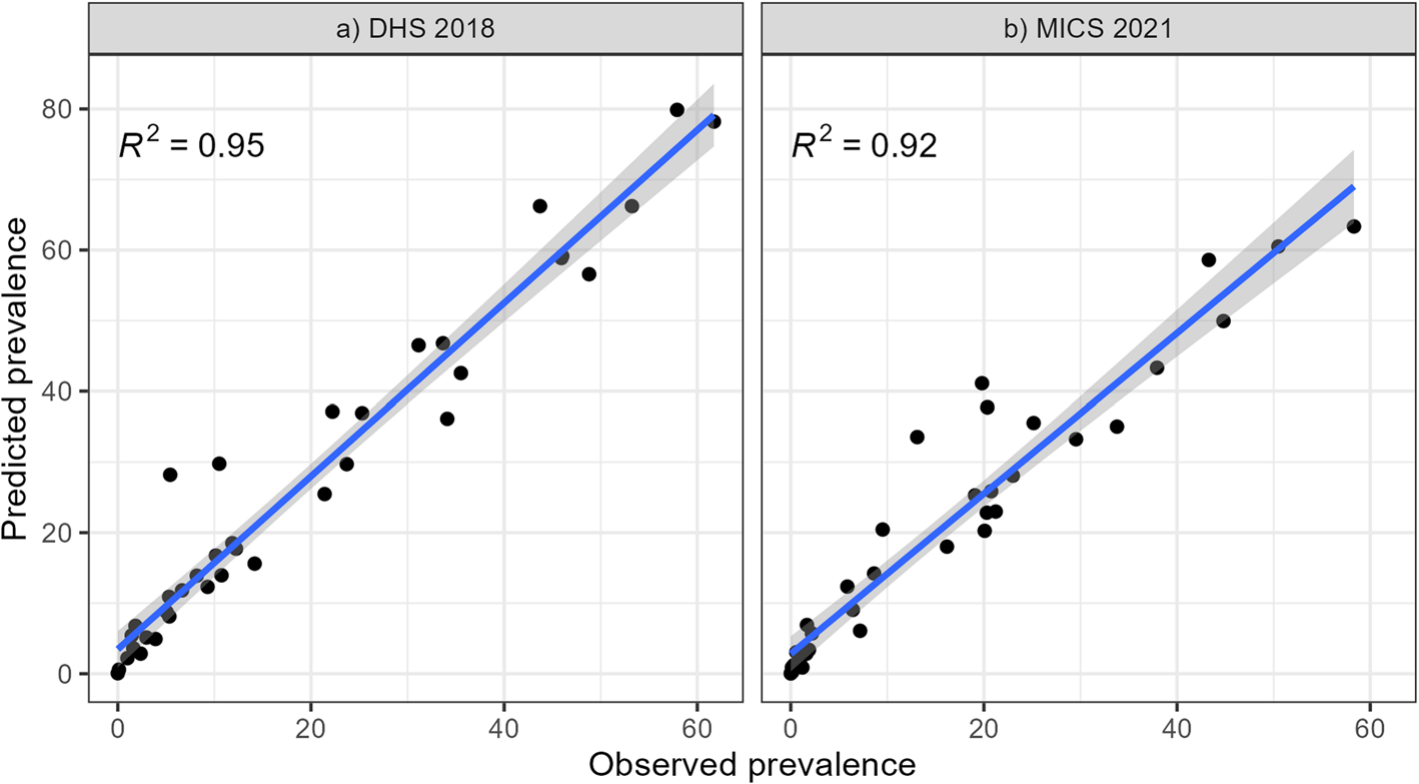
Comparison of observed and predicted FGM prevalence by state in 2018 (**a**) and 2021 (**b**). Posterior estimates are based on the IID models using both individual and community level variables for both DHS 2018 and MICS 2021

The posterior estimates have a low standard deviation, indicating a high level of confidence in the predictions (Fig. 5c and d). Furthermore, Fig. 7 shows a close linear relationship between the observed and predicted prevalence aggregated by state, with high ${R}^{2}$ values (> 0.9) for both the DHS and MICS models. This indicates that the Bayesian framework performs well in the context of modelling the FGM status of women aged 15–49 in Nigeria using the two different datasets. However, it should be noted that the predicted prevalence of FGM at the state level in 2018, while leading to the highest value of ${R}^{2}$, appears to be slightly overestimated compared to the observed prevalence, as most of the points are above the 1:1 line in Fig. 7.

## Discussion

While female genital mutilation (FGM) remains a significant human rights issue, significant progress has been made in recent decades to combat this harmful practice. However, concerns have arisen about the potential setbacks posed by the COVID-19 pandemic [6–8]. The aim of this study was therefore to compare the prevalence and likelihood of FGM among women aged 15–49 in Nigeria, one of the countries with the highest rates of FGM, before and after the COVID-19 pandemic. To achieve this goal, we used Bayesian regression models to analyse the FGM status of women, controlling for individual factors such as marital status, as well as community level factors such as the prevalence of FGM within the community, and geographical location of residence within the state/zone. Our analysis used data from the Demographic and Health Surveys (DHS) conducted in 2018, representing the period prior to COVID-19, and the subsequent 2021 Multiple Indicator Cluster Surveys (MICS), reflecting the post-pandemic landscape.

Analysis of the statistical evidence data shows that the national prevalence of FGM decreased from 25.6% to 17.3% between 2018 and 2021. However, this overall decrease masks regional disparities, with FGM prevalence increasing in some southern states (e.g. Oyo and Abia states) and within the North-Central zone (e.g. Nasarawa and Kwara states) over the same period. The overall decline in FGM at the national level is consistent with the findings of [11], which showed that the prevalence of FGM among Nigerian women aged 15–49 years decreased from 29.6% in 2008 to 18.4% in 2017. However, the spatial patterns were different, with the prevalence of FGM decreasing in the south-eastern states of Nigeria and increasing in the north-western parts of the country over 2003–2017 [11]. Our results show that the opposite occurred over 2018–2021, with an increase in the southern states and north-central Nigeria and a decrease in the northern states. These findings suggest that there may be a potential impact of COVID-19 on these spatio-temporal patterns of FGM as they are consistent with empirical evidence from the *Orchid Project* [10]. This project highlighted the perceived impact of COVID-19 on FGM practice in 14 countries, including Nigeria, through interviews with grassroots activists and local organisations. They reported an increase in the number of girls being cut in south-west Nigeria due to school closures, combined with a lack of prevention and protection hampered by quarantine restrictions [10]. An increase in FGM was also reported in Kwara state and North Central Nigeria, with the re-emergence of socio-cultural norms, a lack of medical supplies and disrupted and reduced health services in these regions [10]. More generally, health services in Nigeria were reported to have been severely curtailed during the pandemic. Some shelters for women and girls at risk of FGM were even closed without alternatives, while there were reports of higher rates of intimate partner violence during the quarantine period [10].

As well as increasing in some Nigerian states over 2018–2021, the prevalence of FGM has also increased among certain ethnic groups, such as the Tiv, Ijaw and Ibibio. In addition, the prevalence of cut women in the community increases the likelihood of FGM after the COVID-19 period more than before, which may be related to the re-emergence of socio-cultural norms as highlighted in [10]. Other results from our Bayesian hierarchical models suggest that after the pandemic, the likelihood of undergoing FGM was significantly lower in wealthier households than in the poorest households. Before the pandemic, however, household wealth had no significant effect on the likelihood of FGM, as previous work has also shown [14, 28]. Increased marriage of girls to earn a bride price has been highlighted as a consequence of the COVID-19 pandemic by previous qualitative studies [7, 8], including in Nigeria [9]. The economic losses caused by the pandemic may have increased wealth heterogeneity between the richest and poorest households, leading the poorest households in particular to marry off their daughters. Increased marriage may also explain why, before the pandemic, the likelihood of FGM was higher among formerly married women than among currently married women, a trend observed in FGM prevalence in Nigeria from 2007 to 2017 by [11], whereas after the pandemic there was no significant difference between these marital statuses. It should be noted, however, that these changes may not be due to an effect of the COVID-19 pandemic, but rather to the evolution of FGM practice and its drivers over time.

Another important finding of this study is that Bayesian spatial regression models always improved model fit compared to non-spatial models using only covariates. Among the spatial models, integrating correlated spatial random effects, to account for spatial autocorrelation between states, did not significantly improve the model’s Deviance Information Criterion (DIC) compared to using uncorrelated (independent and identically distributed) random effects on states. Furthermore, our results show that the best performing models include both individual and community level drivers of FGM. Moreover, models with community level drivers outperform models with individual level drivers. These findings highlight the importance of community influence on individual FGM status and support the social norms theory of FGM practice. Social norms theory is one of the theories advanced to explain why the practice of FGM persists [29–31]. It states that the actions of individuals in a community are influenced not only by their own choices, but also by the social norms of their community, which exert a strong pressure on individuals, with the potential fear of exclusion or persecution by the community if they act contrary [29–31]. Conversely, if it is the community norm to perform FGM, individuals may see it as an opportunity for marriage, peer acceptance and inclusion in the community's social network [31]. It may therefore be difficult for a household to abandon the practice of FGM if it is not in agreement with most community members. Fig. 4c and d support this theory by showing that the likelihood of FGM increases with the prevalence of women cut in the community. We also found that the likelihood of FGM decreased with the ethnic fractionalisation index, suggesting that women in multi-ethnic communities are less at risk of undergoing FGM. Similar results were found for Kenyan girls aged 0–14 years in [18]. Furthermore, we found that the prevalence of FGM increased among women who supported the continuation of the practice during the COVID-19 period. This shows that FGM is still a social norm issue in Nigeria and that it may have been exacerbated by the COVID-19 pandemic.

In terms of key individual level factors, our results showed that the likelihood of FGM was lower among younger women with secondary to higher level education, living in rural areas, and who had never been married or in a union. These findings are consistent with previous studies. For example, [11] also highlighted a lower prevalence of FGM among women with secondary to higher level of education by analysing DHS and MICS data in Nigeria from 2003 to 2017. Similar relationships between FGM and educational attainment were found in a scoping review of FGM in Nigeria [32] and in other countries as well, such as Senegal [33], Chad [34] and more broadly in sub-Saharan Africa [35]. Similar findings have been reported in Nigeria [11, 28], and sub-Saharan Africa [35] regarding the higher likelihood of FGM among women living in urban areas. However, other studies have shown the opposite relationship, with women in rural areas in Senegal being more at risk of FGM in 2005, but less at risk in 2010 [33]. Finally, [28, 35, 36] also found that the likelihood of FGM increased with age and was higher among married women.

This study is the first to assess changes in both FGM likelihood and prevalence before and after the COVID-19 pandemic using multiple data sources while simultaneously controlling for individual and community level characteristics. Several qualitative studies have attempted to understand the perceived impact of COVID-19 through surveys of the population and programme implementers [7–10, 15], but studies which quantified how FGM prevalence has changed over the COVID-19 period at national and sub-national levels are currently lacking. By exploring several Bayesian hierarchical models with both individual and community level drivers, we provide statistical insights into their relationship with a woman's FGM status. Following [14, 18], we have included potential non-linear effects of certain drivers, such as the percentage of women supporting the continuation of FGM or age, leading to a better understanding of their relationship with the likelihood of FGM. Future work could further explore the potential interaction between individual and community level characteristics. In addition, we focussed on FGM prevalence and likelihood in women aged 15–49 years. Further studies could replicate this analysis with girls aged 0–14 years and compare results with other countries to better understand the global impact of the COVID-19 pandemic on FGM practice, both for women and girls.

Nevertheless, this study has several limitations. Although our findings are consistent with empirical evidence from survey research on the impact of COVID-19 on FGM practice, including in Nigeria, changes in FGM prevalence and likelihood may not be due to the COVID-19 pandemic and may simply be due to changes or evolution in the drivers of FGM over time. In addition, the women surveyed in this study could have been cut at any time between their birth and the day before the survey, so there is no certainty that they were cut during the COVID-19 pandemic. Future studies could further investigate the impact of the COVID-19 pandemic on FGM practice by including COVID-19 data in the analyses. Second, we used different types of surveys as a reference before (DHS) and after (MICS) the COVID-19 pandemic, and some differences might exist between the two surveys. Yet, DHS and MICS use a similar sampling design to achieve a representative sample at the sub-national level, thus minimising potential discrepancies in data collection methods. Besides, DHS and MICS have already been used in previous work to study spatio-temporal trends of FGM prevalence in Nigeria [11, 14, 37], and studies showed that trends in FGM likelihood and prevalence were consistent across DHS and MICS. Future research could focus on exploring the differences between these two household surveys and how this affects the accuracy of model parameter estimates. Lastly, by using DHS and MICS data, we rely on self-reporting of FGM status by the women surveyed. This may lead to an underestimation of the true prevalence of FGM, because the practice of FGM has been considered a crime in Nigeria since 2015 [13], and some women may feel reluctant or pressured not to disclose their FGM status to the interviewer. Conversely, social norms may also lead women to falsely report having undergone FGM, either to conform or to avoid repercussions [29].

## Conclusions

In conclusion, our study sheds light on changes in the prevalence and likelihood of female genital mutilation (FGM) among women aged 15–49 years in Nigeria before and after the COVID-19 pandemic. Despite a national decline in FGM prevalence, our findings reveal significant heterogeneity at the sub-national level and by individual/community characteristics. We observed a sharp increase in FGM prevalence in some Nigerian states, such as Nasarawa, while others, such as Kaduna, experienced a significant decline. As the likelihood of FGM increased with the proportion of women who have been cut within the community, the results highlight the ongoing challenge of FGM as a social norm in Nigeria, which may have been exacerbated by the disruption caused by the pandemic. Going forward, policymakers can use the statistical evidence generated by our study to inform targeted interventions aimed at eradicating FGM. Overall, our study highlights the importance of continued monitoring and intervention efforts to combat FGM in Nigeria and beyond.

### Supplementary Information

Additional file 1: Table S1. Observed prevalence of FGM in Nigeria's 36 states and FCT in 2018 (DHS) and 2021 (MICS). Table S2. Posterior odd ratios from the Bayesian models (Base) fitted to DHS 2018 and MICS 2021 data. Table S3. Posterior odd ratios from the Bayesian models (Besag) fitted to DHS 2018 and MICS 2021 data. Table S4. Posterior odd ratios from the Bayesian models (IID + Besag) fitted to DHS 2018 and MICS 2021 data. Fig. S1. Non-linear effects of woman’s age (a,b), percentage cut (c,d) and women supporting FGM continuation (e,f) based on the Base models. Fig. S2. Posterior predicted FGM prevalence among women aged 15-49 years (a,b) and uncertainty (c,d) estimates based on the Base models. Fig. S3. Non-linear effects of woman’s age (a,b), percentage cut (c,d) and women supporting FGM continuation (e,f) based on the Besag models. Fig. S4. Posterior predicted FGM prevalence among women aged 15-49 years (a,b) and uncertainty (c,d) estimates based on the Besag models. Fig. S5. Non-linear effects of woman’s age (a,b), percentage cut (c,d) and women supporting FGM continuation (e,f) based on the IID + Besag models. Fig. S6. Posterior predicted FGM prevalence among women aged 15-49 years (a,b) and uncertainty (c,d) estimates based on the IID + Besag models.

## Data Availability

DHS 2018 and MICS 2021 data for Nigeria can be accessed through the DHS (https://dhsprogram.com/data/dataset_admin/login_main.cfm) and MICS (https://mics.unicef.org/visitors/sign-in) programmes respectively.

## Abbreviations

DHS: Demographic and Health Surveys
DIC: Deviance Information Criterion
EFI: Ethnic fractionalisation index
FCT: Federal Capital Territory
FGM: Female genital mutilation
iCAR: Intrinsic conditional autoregressive
IID: Independent and identically distributed
INLA: Integrated Nested Laplace Approximation
MAE: Mean Absolute Error
MCMC: Markov chain Monte Carlo
MICS: Multiple Indicator Cluster Surveys
POR: Posterior odd ratio
RMSE: Root Mean Square Error
SD: Standard deviation
SDG: Sustainable Development Goal
VAPP Act: Violence against Persons (Prohibition) Act
