# Supplementary material for: Comparison of FGM prevalence among Nigerian women aged 15–49 years using two household surveys conducted before and after the COVID-19 pandemic
Source: BMC Public Health. 2024 Jul 12;24:1866. doi: 10.1186/s12889-024-19069-6 (PMC11241814; doi:10.1186/s12889-024-19069-6)
Supplement: Supplementary file 1 — Additional file 1: Table S1. Observed prevalence of FGM in Nigeria's 36 states and FCT in 2018 (DHS) and 2021 (MICS). Table S2. Posterior odd ratios from the Bayesian models (Base) fitted to DHS 2018 and MICS 2021 data. Table S3. Posterior odd ratios from the Bayesian models (Besag) fitted to DHS 2018 and MICS 2021 data. Table S4. Posterior odd ratios from the Bayesian models (IID + Besag) fitted to DHS 2018 and MICS 2021 data. Fig. S1. Non-linear effects of woman’s age (a,b), percentage cut (c,d) and women supporting FGM continuation (e,f) based on the Base models. Fig. S2. Posterior predicted FGM prevalence among women aged 15-49 years (a,b) and uncertainty (c,d) estimates based on the Base models. Fig. S3. Non-linear effects of woman’s age (a,b), percentage cut (c,d) and women supporting FGM continuation (e,f) based on the Besag models. Fig. S4. Posterior predicted FGM prevalence among women aged 15-49 years (a,b) and uncertainty (c,d) estimates based on the Besag models. Fig. S5. Non-linear effects of woman’s age (a,b), percentage cut (c,d) and women supporting FGM continuation (e,f) based on the IID + Besag models. Fig. S6. Posterior predicted FGM prevalence among women aged 15-49 years (a,b) and uncertainty (c,d) estimates based on the IID + Besag models. [file 12889_2024_19069_MOESM1_ESM.docx]

Supplementary information

**Table S1.** Observed prevalence of FGM in Nigeria's 36 states and FCT in 2018 (DHS) and 2021 (MICS)

| **State** | **DHS 2018 (%)** | **MICS 2021 (%)** | **Difference** |
| --- | --- | --- | --- |
| Abia | 12.2 | 20.3 | 8.1 |
| Adamawa | 0.00 | 0.2 | 0.2 |
| Akwa Ibom | 10.2 | 6.4 | -3.7 |
| Anambra | 21.4 | 13.1 | -8.3 |
| Bauchi | 10.7 | 0.2 | -10.6 |
| Bayelsa | 6.7 | 20.1 | 13.4 |
| Benue | 5.3 | 1.9 | -3.5 |
| Borno | 2.4 | 7.1 | 4.8 |
| Cross River | 11.9 | 23.0 | 11.1 |
| Delta | 3.7 | 33.8 | 0.1 |
| Ebonyi | 53.2 | 20.4 | -32.9 |
| Edo | 35.5 | 29.5 | -6.0 |
| Ekiti | 57.9 | 50.4 | -7.4 |
| Enugu | 25.3 | 19.0 | -6.2 |
| Federal Capital Territory | 5.1 | 1.6 | -3.5 |
| Gombe | 0.1 | 0.0 | -0.1 |
| Imo | 61.7 | 38.0 | -23.7 |
| Jigawa | 34.1 | 1.7 | -32.4 |
| Kaduna | 48.8 | 9.5 | -39.3 |
| Kano | 22.2 | 19.8 | -2.4 |
| Katsina | 1.4 | 5.9 | 4.4 |
| Kebbi | 1.6 | 0.6 | -1.0 |
| Kogi | 1.0 | 0.9 | -0.1 |
| Kwara | 46.0 | 58.3 | 12.3 |
| Lagos | 23.7 | 21.2 | -2.5 |
| Nasarawa | 1.8 | 20.7 | 18.9 |
| Niger | 10.5 | 0.7 | -9.8 |
| Ogun | 8.2 | 8.6 | 0.5 |
| Ondo | 43.7 | 25.1 | -18.6 |
| Osun | 45.9 | 44.7 | -1.1 |
| Oyo | 31.1 | 43.2 | 12.1 |
| Plateau | 3.0 | 0.9 | -2.1 |
| Rivers | 9.3 | 16.2 | 6.9 |
| Sokoto | 5.4 | 2.2 | -3.2 |
| Taraba | 3.9 | 0.3 | -3.6 |
| Yobe | 14.2 | 1.2 | -13.0 |
| Zamfara | 5.3 | 0.1 | -5.2 |

*Note. “*Difference” column indicates the difference between the FGM prevalence in 2021 and 2018.

**Table S2.** Posterior odd ratios from the Bayesian models (Base) fitted to DHS 2018 and MICS 2021 data.

| Variables | Levels | DHS 2018 | | | MICS 2021 | | |
| --- | --- | --- | --- | --- | --- | --- | --- |
|  |  | **POR** | **2.5%** | **97.5%** | **POR** | **2.5%** | **97.5%** |
|  | (Intercept) | 2.208 | 1.561 | 3.125 | 1.610 | 1.111 | 2.330 |
| Geopolitical zone | North-North (ref) | 1 | - | - | 1 | - | - |
|  | North-East | 0.378 | 0.281 | 0.508 | 0.445 | 0.277 | 0.715 |
|  | North-West | 0.571 | 0.444 | 0.735 | 1.199 | 0.914 | 1.574 |
|  | South-East | 0.587 | 0.445 | 0.774 | 0.671 | 0.524 | 0.861 |
|  | South-South | 0.663 | 0.512 | 0.858 | 0.709 | 0.559 | 0.900 |
|  | South-West | 0.916 | 0.724 | 1.159 | 0.866 | 0.691 | 1.086 |
| Residence | Rural (ref) | 1 | - | - | 1 | - | - |
|  | Urban | 0.990 | 0.861 | 1.139 | 1.186 | 1.024 | 1.373 |
| Education | No education (ref) | 1 | - | - | 1 | - | - |
|  | Higher | 0.630 | 0.510 | 0.779 | 0.667 | 0.543 | 0.818 |
|  | Primary | 1.169 | 0.989 | 1.380 | 1.033 | 0.866 | 1.233 |
|  | Secondary | 0.826 | 0.698 | 0.978 | 0.783 | 0.659 | 0.931 |
| Age |  | *See Figure S1a* | | | *See Figure S1b* | | |
| Wealth quintile | Poorest (ref) | 1 | - | - | 1 | - | - |
|  | Poorer | 1.022 | 0.859 | 1.217 | 0.888 | 0.746 | 1.058 |
|  | Middle | 0.983 | 0.813 | 1.188 | 0.874 | 0.731 | 1.046 |
|  | Richer | 0.940 | 0.765 | 1.154 | 0.765 | 0.631 | 0.927 |
|  | Richest | 0.957 | 0.763 | 1.200 | 0.602 | 0.486 | 0.746 |
| Marital status | Currently married/in union (ref) | 1 | - | - | 1 | - | - |
|  | Formerly married/in union | 1.430 | 1.174 | 1.741 | 1.049 | 0.894 | 1.231 |
|  | Never married/in union | 0.665 | 0.575 | 0.771 | 0.597 | 0.516 | 0.691 |
| Percentage women cut |  | *See Figure S1c* | | | *See Figure S1d* | | |
| Percentage women supporting FGM continuation |  | *See Figure S1e* | | | *See Figure S1f* | | |
| EFI |  | 0.586 | 0.426 | 0.806 | 0.795 | 0.588 | 1.075 |
| Main religion in community | Christian (ref) | 1 | - | - | 1 | - | - |
|  | Islam | 0.938 | 0.765 | 1.150 | 1.130 | 0.941 | 1.356 |
|  | Traditional | 0.372 | 0.041 | 3.346 | 0.000 | 0.000 | 7.726 |
| Sampling weight |  | 0.989 | 0.903 | 1.083 | 1.003 | 0.961 | 1.047 |

*Note.* Posterior odd ratios (POR) estimates are based on the Base models using both individual and community level variables for both DHS 2018 and MICS 2021. Underlined figures indicate significant relationships, i.e. when the 2.5% and 97.5% CIs are both either greater or less than 1.


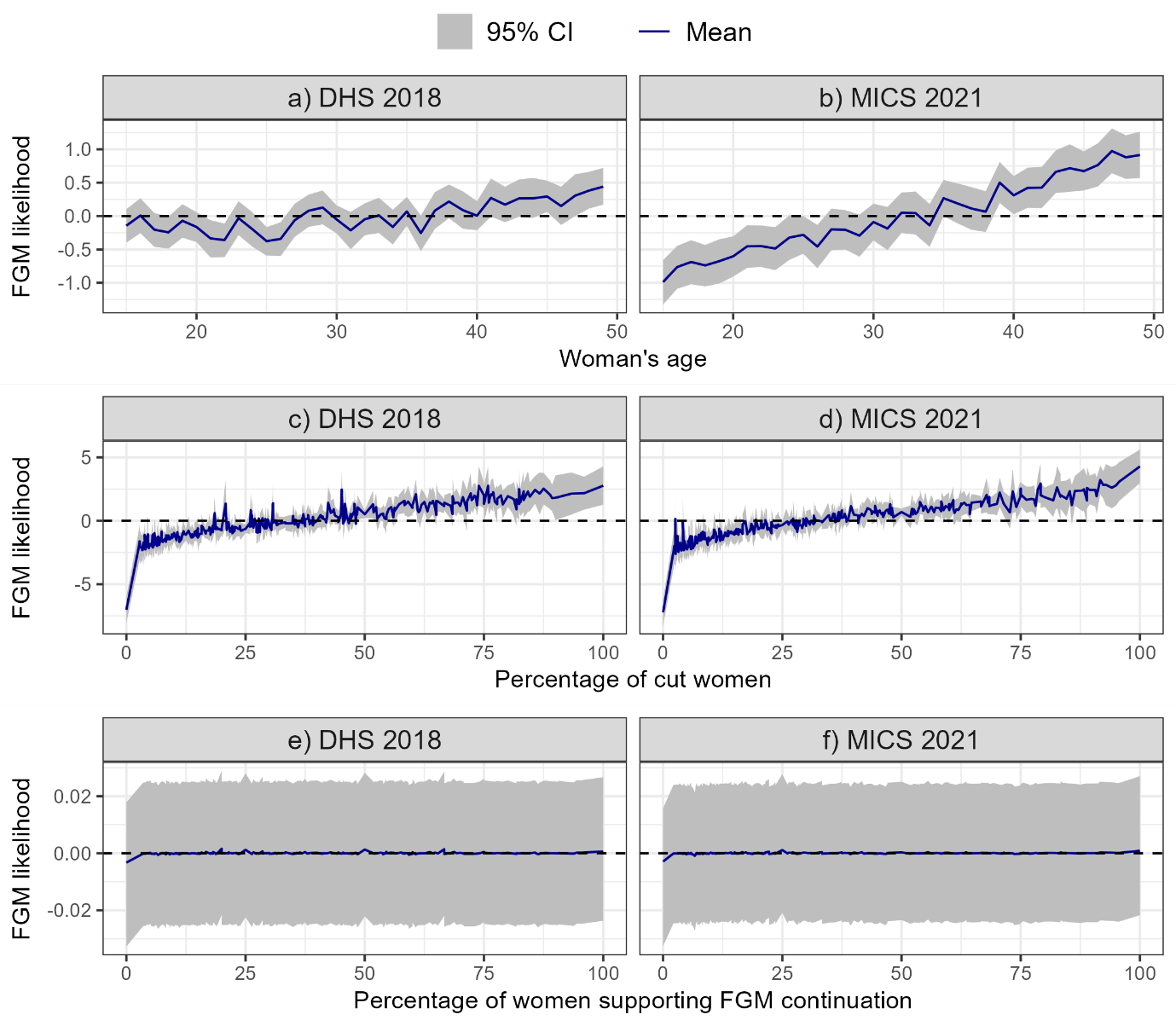


**Figure S1.** Non-linear effects of woman’s age (a,b), percentage cut (c,d) and women supporting FGM continuation (e,f) based on the Base models. Estimates are based on the Base models using both individual and community level variables for both DHS 2018 and MICS 2021.

**
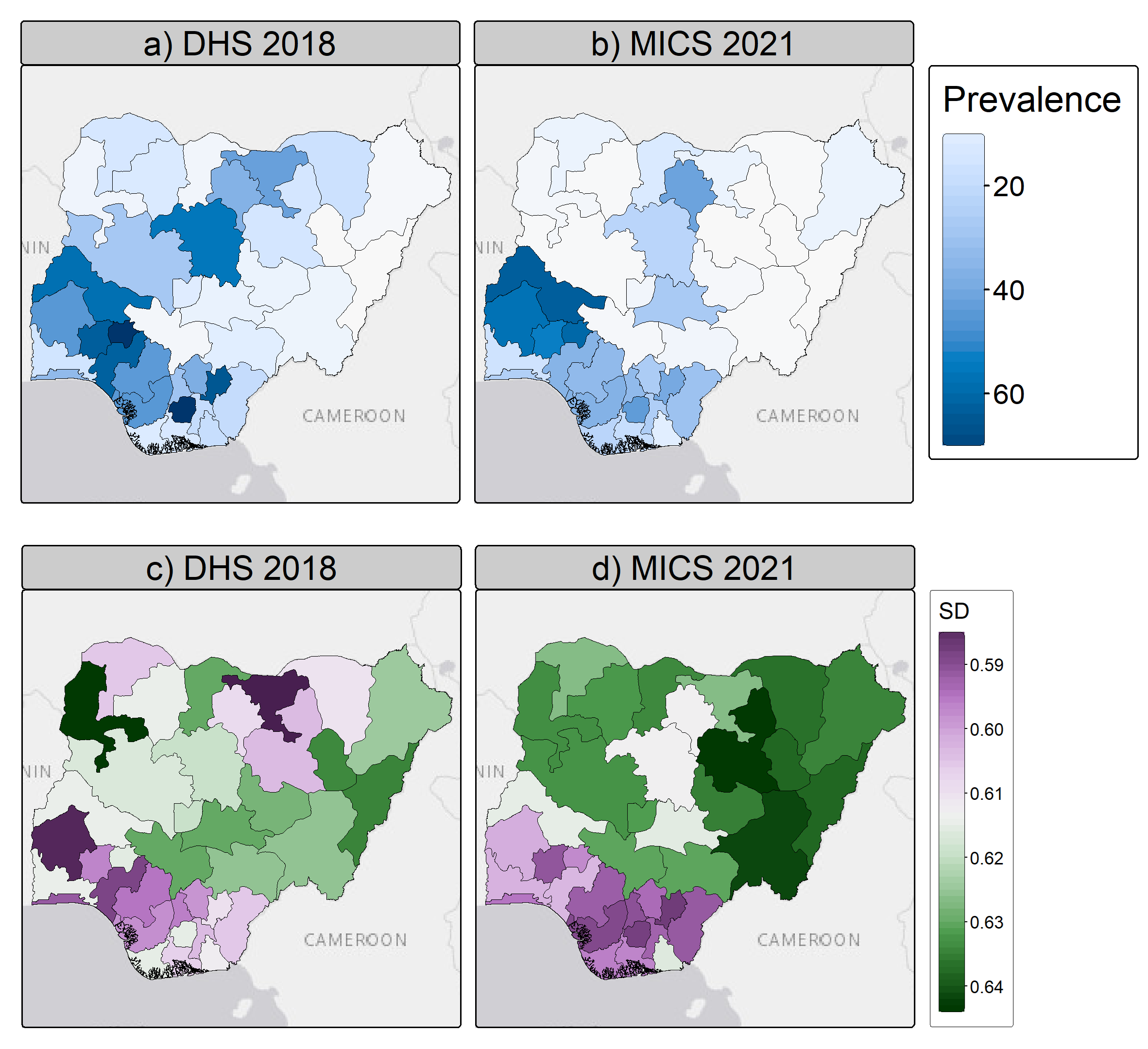
**

**Figure S2.** Posterior predicted FGM prevalence among women aged 15-49 years (a,b) and uncertainty (c,d) estimates based on the Base models. Posterior estimates are based on the Base models using both individual and community level variables for both DHS 2018 and MICS 2021. SD stands for standard deviation. Shapefile downloaded from GADM.

**Table S3.** Posterior odd ratios from the Bayesian models (Besag) fitted to DHS 2018 and MICS 2021 data.

| Variables | Levels | DHS 2018 | | | MICS 2021 | | |
| --- | --- | --- | --- | --- | --- | --- | --- |
|  |  | **POR** | **2.5%** | **97.5%** | **POR** | **2.5%** | **97.5%** |
|  | (Intercept) | 1.905 | 1.154 | 3.131 | 1.558 | 1.028 | 2.357 |
| Geopolitical zone | North-North (ref) | 1 | - | - | 1 | - | - |
|  | North-East | 0.409 | 0.179 | 0.926 | 0.400 | 0.200 | 0.774 |
|  | North-West | 0.627 | 0.304 | 1.284 | 1.262 | 0.816 | 1.971 |
|  | South-East | 0.905 | 0.403 | 2.114 | 0.730 | 0.473 | 1.145 |
|  | South-South | 0.957 | 0.424 | 2.248 | 0.767 | 0.499 | 1.203 |
|  | South-West | 1.438 | 0.660 | 3.242 | 0.915 | 0.613 | 1.383 |
| Residence | Rural (ref) | 1 | - | - | 1 | - | - |
|  | Urban | 0.997 | 0.858 | 1.158 | 1.169 | 1.006 | 1.360 |
| Education | No education (ref) | 1 | - | - | 1 | - | - |
|  | Higher | 0.584 | 0.471 | 0.725 | 0.667 | 0.543 | 0.820 |
|  | Primary | 1.123 | 0.948 | 1.330 | 1.050 | 0.879 | 1.253 |
|  | Secondary | 0.781 | 0.658 | 0.927 | 0.790 | 0.664 | 0.939 |
| Age |  | *See Figure S3a* | | | *See Figure S3b* | | |
| Wealth quintile | Poorest (ref) | 1 | - | - | 1 | - | - |
|  | Poorer | 0.914 | 0.763 | 1.093 | 0.890 | 0.746 | 1.061 |
|  | Middle | 0.859 | 0.706 | 1.046 | 0.858 | 0.714 | 1.030 |
|  | Richer | 0.824 | 0.664 | 1.022 | 0.740 | 0.607 | 0.903 |
|  | Richest | 0.869 | 0.685 | 1.103 | 0.587 | 0.471 | 0.732 |
| Marital status | Currently married/in union (ref) | 1 | - | - | 1 | - | - |
|  | Formerly married/in union | 1.440 | 1.181 | 1.755 | 1.055 | 0.899 | 1.238 |
|  | Never married/in union | 0.654 | 0.565 | 0.759 | 0.598 | 0.517 | 0.692 |
| Percentage women cut |  | *See Figure S3c* | | | *See Figure S3d* | | |
| Percentage women supporting FGM continuation |  | *See Figure S3e* | | | *See Figure S3f* | | |
| EFI |  | 0.635 | 0.449 | 0.897 | 0.833 | 0.608 | 1.142 |
| Main religion in community | Christian (ref) | 1 | - | - | 1 | - | - |
|  | Islam | 0.953 | 0.765 | 1.188 | 1.084 | 0.891 | 1.317 |
|  | Traditional | 0.571 | 0.058 | 5.621 | 0.000 | 0.000 | 2.252 |
| Sampling weight |  | 0.993 | 0.902 | 1.094 | 0.997 | 0.952 | 1.044 |

*Note.* Posterior odd ratios (POR) estimates are based on the Besag models using both individual and community level variables for both DHS 2018 and MICS 2021. Underlined figures indicate significant relationships, i.e. when the 2.5% and 97.5% CIs are both either greater or less than 1.


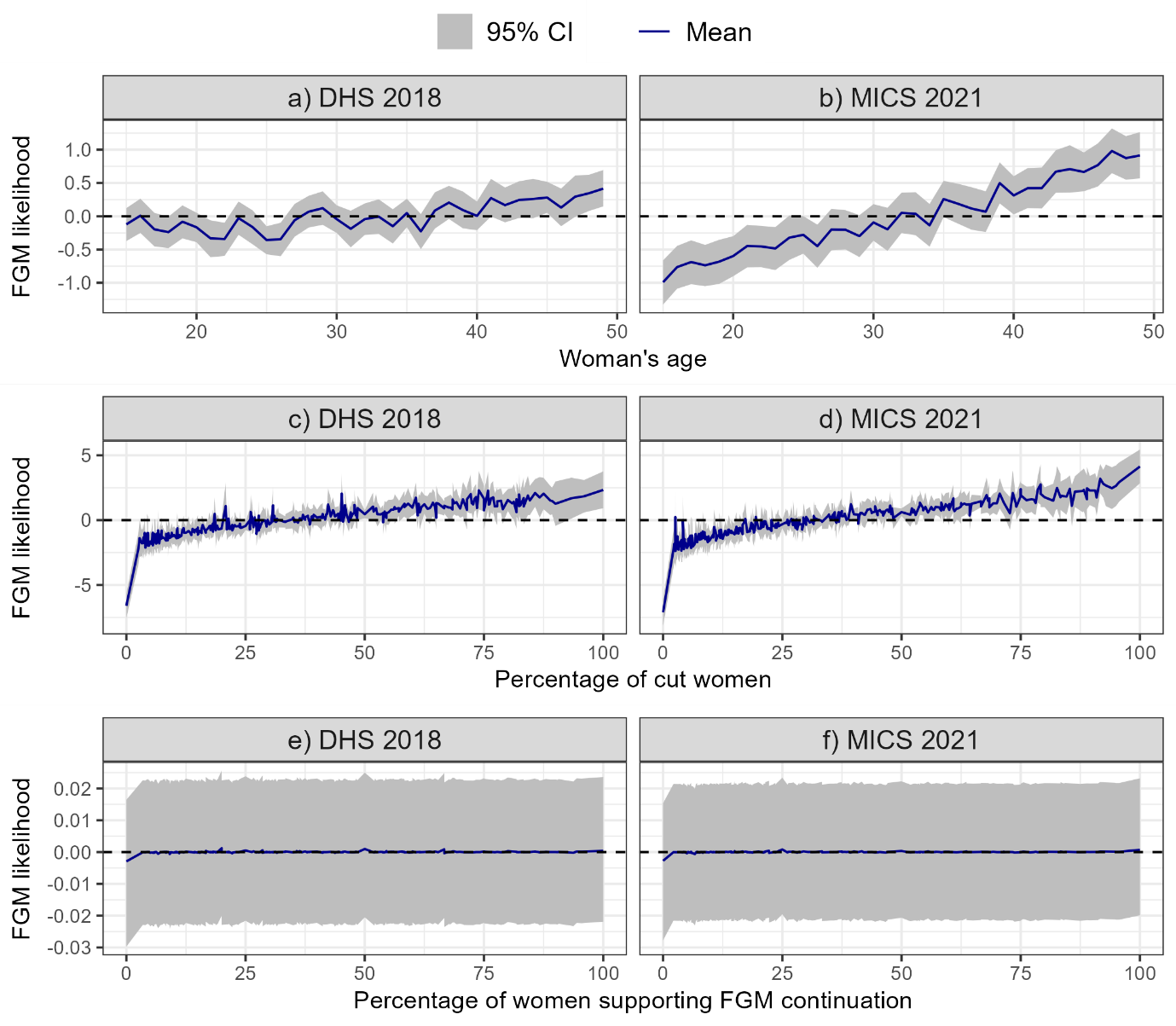


**Figure S3.** Non-linear effects of woman’s age (a,b), percentage cut (c,d) and women supporting FGM continuation (e,f) based on the Besag models. Estimates are based on the Besag models using both individual and community level variables for both DHS 2018 and MICS 2021.


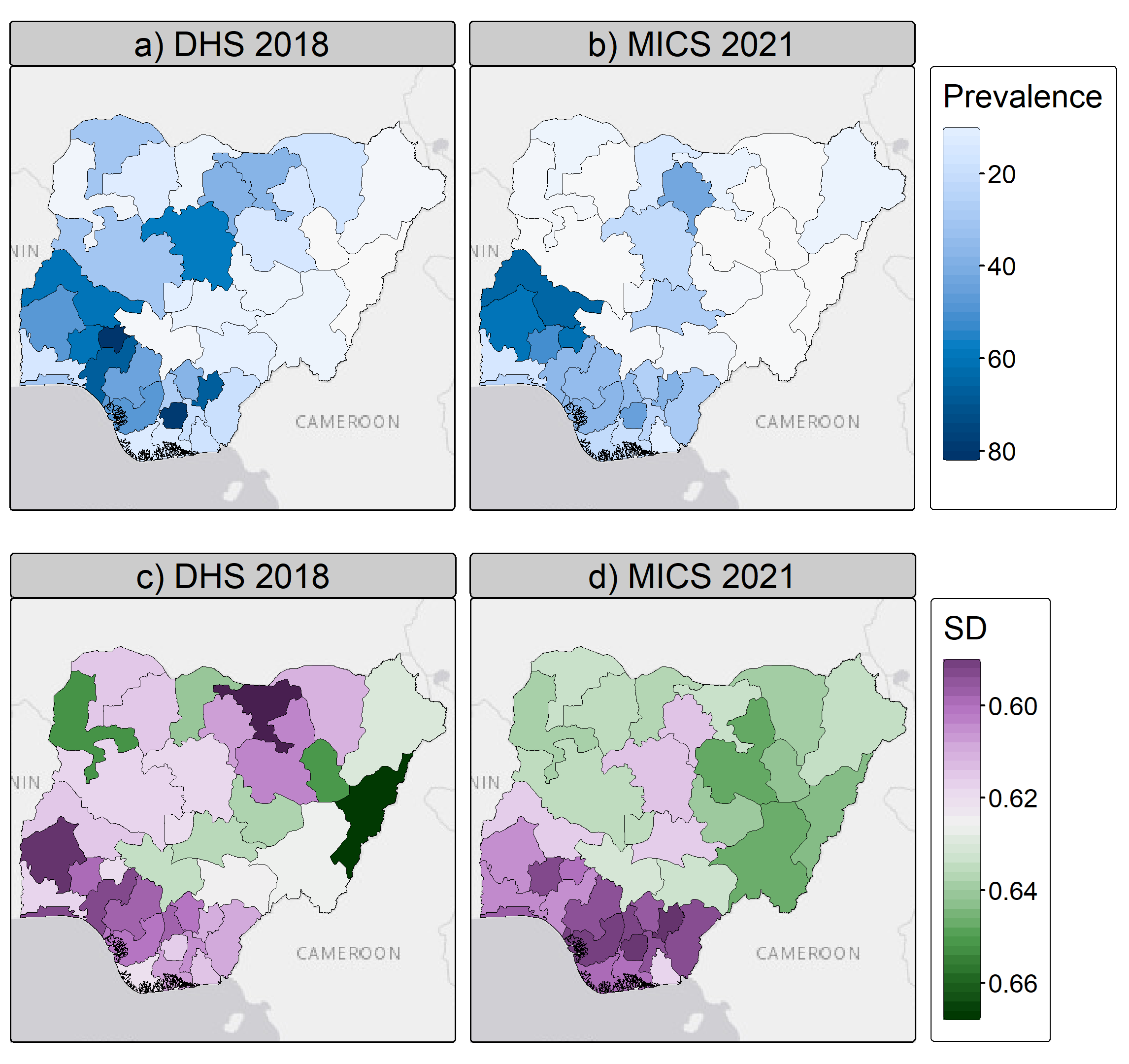


**Figure S4.** Posterior predicted FGM prevalence among women aged 15-49 years (a,b) and uncertainty (c,d) estimates based on the Besag models. Posterior estimates are based on the Besag models using both individual and community level variables for both DHS 2018 and MICS 2021. SD stands for standard deviation. Shapefile downloaded from GADM.

**Table S4.** Posterior odd ratios from the Bayesian models (IID + Besag) fitted to DHS 2018 and MICS 2021 data.

| Variables | Levels | DHS 2018 | | | MICS 2021 | | |
| --- | --- | --- | --- | --- | --- | --- | --- |
|  |  | **POR** | **2.5%** | **97.5%** | **POR** | **2.5%** | **97.5%** |
|  | (Intercept) | 2.036 | 1.239 | 3.333 | 1.489 | 0.978 | 2.249 |
| Geopolitical zone | North-North (ref) | 1 | - | - | 1 | - | - |
|  | North-East | 0.399 | 0.211 | 0.757 | 0.456 | 0.265 | 0.787 |
|  | North-West | 0.715 | 0.409 | 1.261 | 1.354 | 0.942 | 1.995 |
|  | South-East | 0.783 | 0.431 | 1.443 | 0.736 | 0.524 | 1.055 |
|  | South-South | 0.789 | 0.449 | 1.401 | 0.771 | 0.556 | 1.089 |
|  | South-West | 1.220 | 0.700 | 2.159 | 0.989 | 0.718 | 1.394 |
| Residence | Rural (ref) | 1 | - | - | 1 | - | - |
|  | Urban | 1.001 | 0.862 | 1.163 | 1.173 | 1.010 | 1.363 |
| Education | No education (ref) | 1 | - | - | 1 | - | - |
|  | Higher | 0.583 | 0.470 | 0.724 | 0.669 | 0.544 | 0.822 |
|  | Primary | 1.120 | 0.946 | 1.327 | 1.052 | 0.881 | 1.256 |
|  | Secondary | 0.779 | 0.657 | 0.925 | 0.792 | 0.666 | 0.942 |
| Age |  | *See Figure S5a* | | | *See Figure S5b* | | |
| Wealth quintile | Poorest (ref) | 1 | - | - | 1 | - | - |
|  | Poorer | 0.908 | 0.758 | 1.086 | 0.891 | 0.747 | 1.063 |
|  | Middle | 0.852 | 0.700 | 1.038 | 0.864 | 0.720 | 1.038 |
|  | Richer | 0.817 | 0.659 | 1.013 | 0.749 | 0.614 | 0.912 |
|  | Richest | 0.860 | 0.678 | 1.091 | 0.593 | 0.477 | 0.739 |
| Marital status | Currently married/in union (ref) | 1 | - | - | 1 | - | - |
|  | Formerly married/in union | 1.439 | 1.181 | 1.755 | 1.055 | 0.899 | 1.238 |
|  | Never married/in union | 0.651 | 0.562 | 0.755 | 0.598 | 0.516 | 0.692 |
| Percentage women cut |  | *See Figure S5c* | | | *See Figure S5d* | | |
| Percentage women supporting FGM continuation |  | *See Figure S5e* | | | *See Figure S5f* | | |
| EFI |  | 0.619 | 0.438 | 0.874 | 0.814 | 0.595 | 1.113 |
| Main religion in community | Christian (ref) | 1 | - | - | 1 | - | - |
|  | Islam | 0.951 | 0.763 | 1.185 | 1.099 | 0.905 | 1.333 |
|  | Traditional | 0.566 | 0.058 | 5.523 | 0.000 | 0.000 | 1.778 |
| Sampling weight |  | 0.986 | 0.896 | 1.085 | 0.996 | 0.951 | 1.042 |

*Note.* Posterior odd ratios (POR) estimates are based on the IID + Besag models using both individual and community level variables for both DHS 2018 and MICS 2021. Underlined figures indicate significant relationships, i.e. when the 2.5% and 97.5% CIs are both either greater or less than 1.


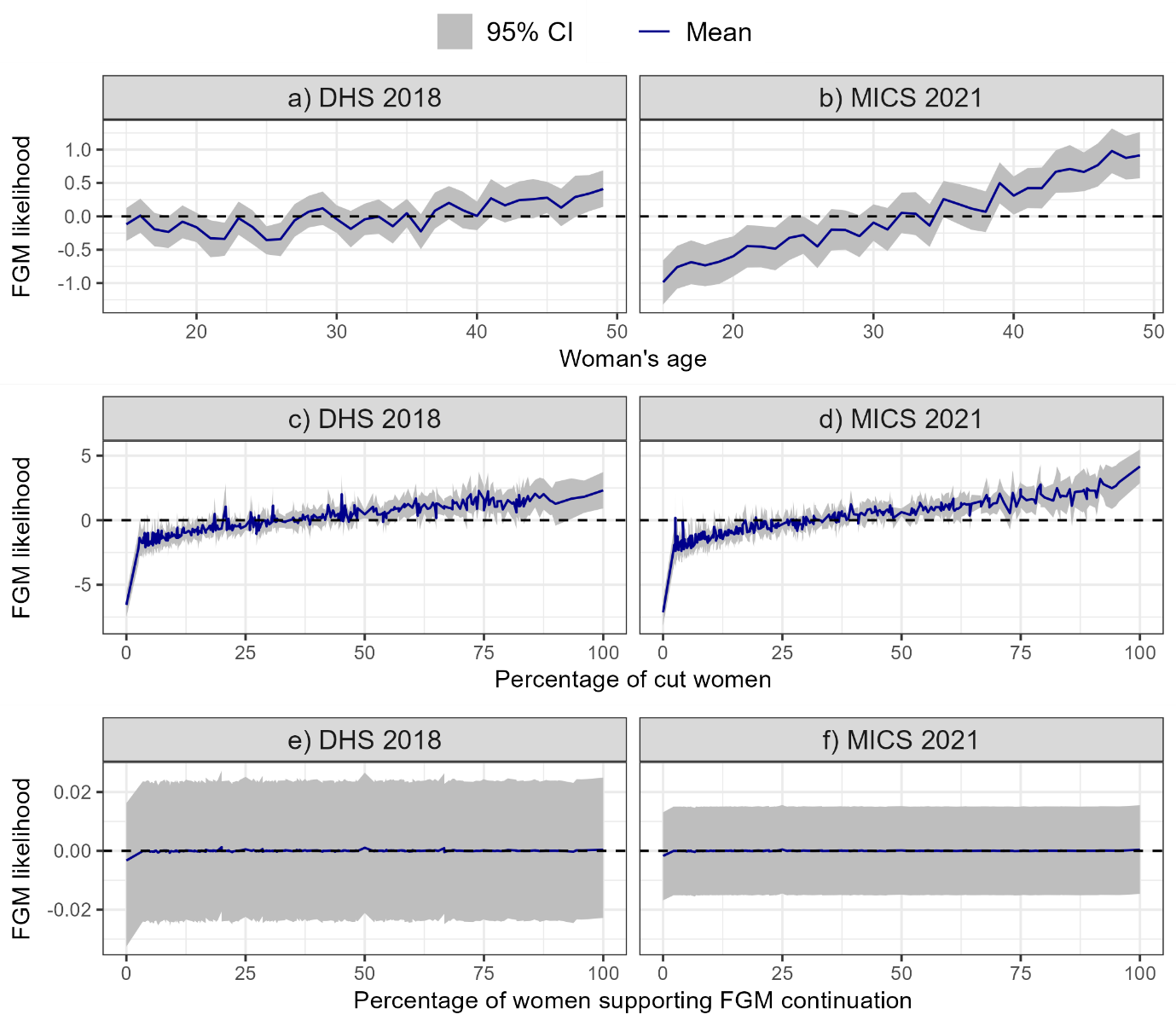


**Figure S5.** Non-linear effects of woman’s age (a,b), percentage cut (c,d) and women supporting FGM continuation (e,f) based on the IID + Besag models. Estimates are based on the IID + Besag models using both individual and community level variables for both DHS 2018 and MICS 2021.


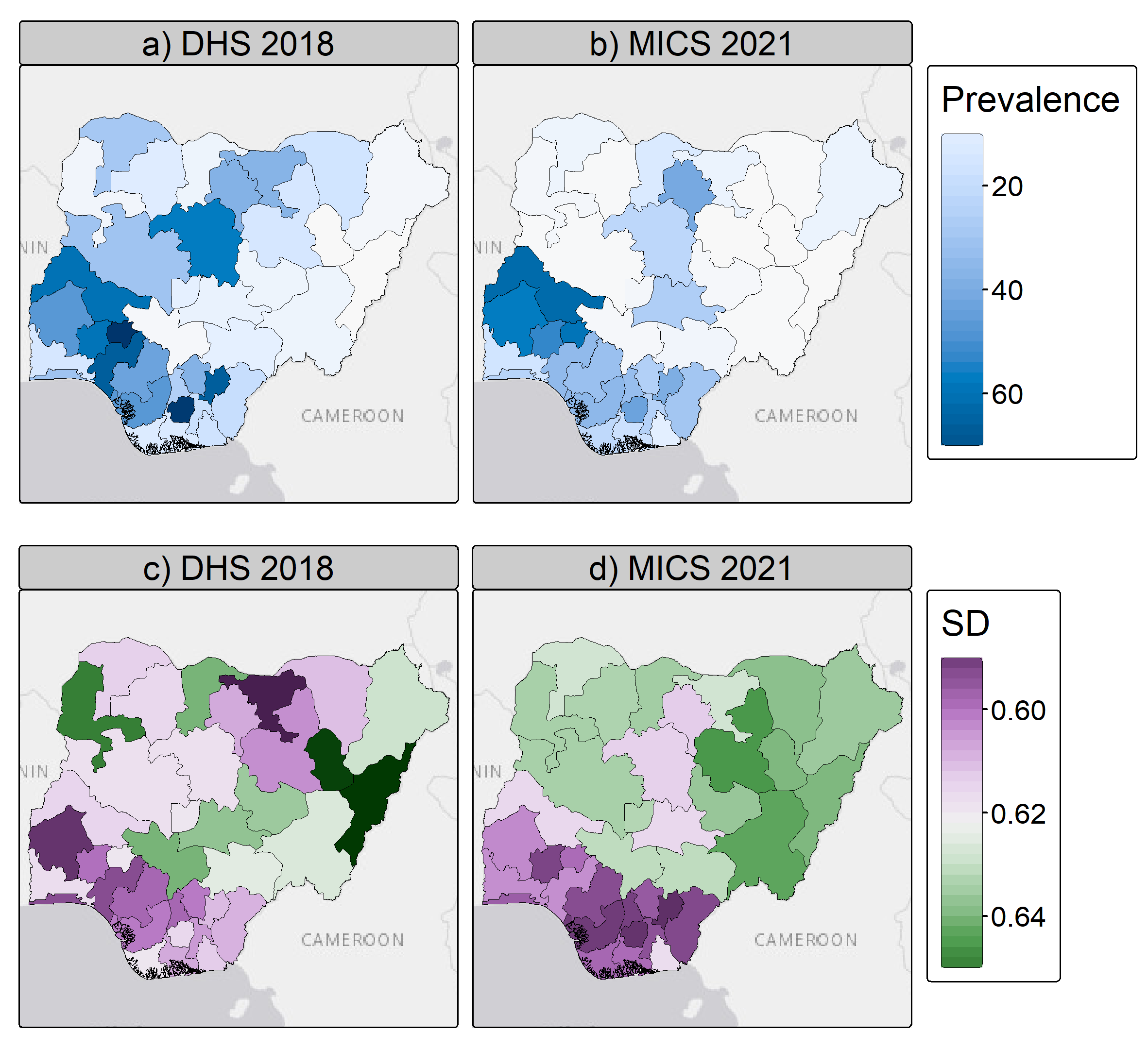


**Figure S6.** Posterior predicted FGM prevalence among women aged 15-49 years (a,b) and uncertainty (c,d) estimates based on the IID + Besag models. Posterior estimates are based on the IID + Besag models using both individual and community level variables for both DHS 2018 and MICS 2021. SD stands for standard deviation. Shapefile downloaded from GADM.
